# The mechanism of gut microbiota in septic cardiomyopathy based on the bulk transcriptome and Mendelian randomization analysis

**DOI:** 10.3389/fimmu.2026.1799675

**Published:** 2026-05-04

**Authors:** Yuxia Tao, Lianxin Li, Jianhao Wang, Wenting Du, Shan Ye, Jinshuai Lu

**Affiliations:** Emergency Center II, People’s Hospital of Xinjiang Uygur Autonomous Region, Urumqi, China

**Keywords:** biomarkers, gut microbiota, machine learning, Mendelian randomization, septic cardiomyopathy

## Abstract

**Background:**

Septic cardiomyopathy (SCM) is one of the most common and serious complications of sepsis. Earlier research has identified reciprocal regulation between SCM and gut microbiota (GM), but the mechanistic basis of GM in SCM development remains obscure. This study aimed to explore the value of targets of GM in SCM.

**Methods:**

The genome-wide association study (GWAS) data of sepsis and GM were downloaded from public databases. The causal association between GM and sepsis was evaluated via Mendelian randomization (MR) analysis to identify key microbiota potentially involved in septic complications. The metabolites and targets corresponding to the GM were acquired from the public databases. The differentially expressed genes (DEGs) between SCM and control were obtained by differential expression analysis. Subsequently, biomarkers were identified by the intersection of DEGs and targets of GM, and machine learning algorithm. Gene set enrichment analysis (GSEA), and immune analysis were adopted. Then, the upstream factors and metabolites linked to biomarkers were obtained. Finally, molecular docking and molecular dynamics (MD) simulation analysis was conducted. Additionally, the concentrations of the inflammatory cytokine IL-6 and the myocardial injury marker cTnI in clinical samples were detected using enzyme-linked immunosorbent assay (ELISA). The concentration of the key metabolite propylene glycol was measured via gas chromatography-mass spectrometry (GC-MS). The expression levels of biomarker genes were validated through reverse transcription quantitative polymerase chain reaction (RT-qPCR).

**Results:**

A total of 5 GM, such as genus.Bifidobacterium.id.436.scatter were obtained based on MR analysis, and 22 metabolites, such as folic acid, and 461 targets, such as CA12 were obtained. Then, a total of 166 DEGs were determined, a total of 11 candidate genes were obtained through the intersection of the targets of GM and SCM. Finally, STAT3 and SLC5A1 were identified as biomarkers, and these 2 genes were both enriched in the pathways such as valine leucine and isoleucine degradation. Moreover, immune cells such as MDSCs and activated dendritic cells might have a notable impact on SCM with biomarkers. Transcription factors (TFs) such as FOXC1 and microRNAs (miRNAs) such as miR-3120-3p have been found to have regulatory relationships with biomarkers, and propylene glycol had good binding activity with biomarkers. Finally, clinical sample validation results demonstrated that serum IL-6 and cTnI concentrations were significantly elevated in SCM patients, while propylene glycol levels were markedly decreased. Concurrently, STAT3 mRNA expression was significantly upregulated, whereas SLC5A1 expression was significantly downregulated.

**Conclusion:**

This study identified STAT3 and SLC5A1 as candidate biomarkers associated with gut microbiota in SCM, providing a foundation for future investigation into their mechanistic roles.

## 1 Introduction

Sepsis is a serious condition that causes the body to have a severe and uncontrolled immune reaction to an infection (1). Multiple organ dysfunction is the leading cause of death in patients with sepsis, with the heart being one of the most commonly affected organs. It has been documented that between 20% and 50% of patients diagnosed with sepsis also exhibit myocardial injury, and their mortality rate is considerably higher in comparison to patients with sepsis without myocardial injury (2, 3). SCM is characterized by ventricular dilatation, reduced ejection fraction, and impaired contractility. The pathogenesis of this condition is intricate and multifactorial, involving primarily dysregulated inflammatory responses, oxidative stress, mitochondrial dysfunction, myocardial cell apoptosis, calcium ion imbalance, and autonomic nervous system dysfunction (4). Despite contemporary diagnostic techniques, including echocardiography and cardiac magnetic resonance imaging, which have facilitated significant advancements in the diagnosis of SCM (5), the application of these techniques in the diagnosis and monitoring of SCM remains challenging. Despite extensive research on the treatment of septic cardiomyopathy, no effective drug treatment regimen has yet been established for this condition (6). Consequently, the identification of novel diagnostic biomarkers and therapeutic targets for SCM is imperative for the early diagnosis of the condition, the elucidation of its pathogenesis, and the enhancement of patient prognosis.

The GM is defined as the complex microbial community residing in the gastrointestinal tract, comprising bacteria, archaea, fungi, and viruses. These microorganisms play a pivotal role in affecting the health status of the host (7). The intestinal tract is believed to play an important role in the pathogenesis of sepsis and sepsis-induced multiple organ dysfunction. The composition of the GM is influenced by sepsis, and disruption of the GM may contribute to the development of organ failure (8). A genomic analysis of the GM in patients with sepsis reveals that microbial dysbiosis is associated with sepsis-induced organ damage, and microbial changes is related to sepsis progression (9). In contrast with the control group, patients with sepsis displayed aberrations in intestinal amino acid metabolism, including tryptophan metabolism. These abnormalities were found to be closely associated with changes in the severity of sepsis (10). The GM has been demonstrated to play a pivotal role in the development of sepsis and various cardiovascular diseases, for instance, coronary heart disease (11), heart failure, and myocardial infarction (12). Research has demonstrated that the GM can enhance myocardial injury through the regulation of metabolite levels and the modulation of related target genes (13). The application of probiotic therapy has been found to markedly reduce the incidence of sepsis-induced cardiac injury in a murine model of sepsis (14). In the event of sepsis, alterations in the GM may contribute to the severity of the condition by facilitating the dissemination of pathogens, inducing immune dysregulation, and diminishing the production of beneficial short-chain fatty acids (15). Disruption of the GM has the potential to indirectly induce SCM by triggering key pathological factors of sepsis, such as the systemic inflammatory response, oxidative stress, endothelial dysfunction, and so forth. Moreover, it may also directly affect myocardial damage through regulating mitochondrial function, calcium signaling pathways, and myocardial cell apoptosis (16). However, the causal relationship between GM and SCM remains unclear, as does the specific mechanisms underlying this association. A comprehensive investigation into the mechanisms by which GM influence SCM may facilitate the identification of novel therapeutic targets for this disease.

MR analysis employs genetic variation as an instrumental variable (IV) for the exposure of interest, thereby providing an analytical method that approximates the causal relationship between a specific exposure and disease outcomes. In comparison with conventional methods, MR analysis effectively addresses the issue of confounding factors in traditional observational studies. This enables more accurate inference of causal relationships and provides a solid foundation for the subsequent formulation of precise disease prevention strategies and evaluation of intervention measures (17). MR analysis is a widely utilized approach in diverse fields, including cancer research, obstetrics and gynecology, pharmacology, and public health. Its applications encompass the exploration of etiology, the identification of novel drug targets, and the characterization of biomarkers. Consequently, the present study elected to use MR as a point of departure to investigate the relationship between GM and sepsis, and through further integration of SCM transcriptomic data, the study aims to elucidate the potential molecular mechanisms by which the GM influences the progression of SCM.

The present study is a public database-based study because there are currently no GWAS data related to SCM in public databases, and SCM is one of the common complications of sepsis. Therefore, we selected sepsis-related GWAS data, gut microbiota-related GWAS data, and SCM transcriptomic data. By using MR analysis, we were able to identify key GM with significant causal relationships with sepsis. We then used the gutMGene v1.0 database to identify target genes corresponding to the key GM. We intersected them with differentially expressed genes from the SCM transcriptomics dataset to obtain candidate genes. Finally, we screened for key biomarkers using machine learning. Finally, we analyzed the potential molecular mechanisms of GM-related genes as biomarkers for SCM through a series of bioinformatics methods. The objective of this study is to conduct a preliminary investigation into the potential molecular mechanisms underlying the role of the gut microbiota in the onset and progression of SCM. Thereby establish novel research directions for the early diagnosis, pathogenesis, and treatment of SCM.

## 2 Methods

### 2.1 Data collection

The transcriptome data (GSE79962) of septic cardiomyopathy (SCM) were retrieved from the Gene Expression Omnibus (GEO) database (https://www.ncbi.nlm.nih.gov/geo/). The GSE79962 dataset (GPL6244) includes 20 SCM and 11 control tissue samples. The information of metabolites related to gut microbiota (GM) was sourced from the gutMGene database (https://ngdc.cncb.ac.cn/databasecommons/database/). The ieu-b-4980 of sepsis was retrieved from the Catalog Open GWAS database (https://www.ebi.ac.uk/gwas/), with a sample size of 486, 484, including 12, 243, 539 single nucleotide polymorphisms (SNPs) from 11, 643 cases and 474, 841 controls of Europeans. The data of GM were downloaded from the GWAS database, including 16S ribosomal RNA gene sequencing profiles and genotyping data of a total of 18340 participants from 24 cohorts in 11 countries (the United States, South Korea, Canada, Israel, Germany, Denmark, the Netherlands, Finland, Belgium, Sweden, and the United Kingdom).

### 2.2 MR analysis

To investigate the causal relationship between GM and sepsis, an MR analysis via the “TwoSampleMR” package (v 0.6.14) (18)was adopted. In MR analysis, GM was regarded as exposure factors and sepsis was regarded as outcome, respectively. Then, the instrumental variables (IVs) notably linked to GM (P < 5 × 10^-8^) were reserved, and the thresholds of linkage disequilibrium (LD) were clump = TRUE, R^2^ < 0.001, and kb = 10. IVs significantly correlated with the outcome variable were excluded (proxies=TRUE, rsq=0.8). Then, the number of SNPs was greater than 3, and the SNPs with F-values greater than 10 were reserved. The calculation formula for F values was as follows, and R^2^ represents the cumulative explanatory variance of the selected SNPs, and N represents the number of samples, and K represents the number of SNPs in the formula.


$$F=\frac{R^{2}\;(n\;-\;K-\;1)}{K\;(1\;-\;R^{2})}$$

In addition, SNPs with palindrome sequences need to be excluded. Finally, the MR function was utilized to harmonize the effect alleles and effect sizes, and MR Egger (19), Weighted median (20), Inverse variance weighted (IVW) (21), Simple mode, and Weighted mode were utilized to execute MR analysis. Then, analyze between the obtained exposure factors and sepsis were performed separately, and the exposure factors with P < 0.05 were selected as potential exposure factors for subsequent analysis based on the results of IVW, while a threshold was not set in the other methods. The results were pictured via the “forestplot” package (v 3.1.6) (22). Then, the correlation between potential exposure factors and sepsis was visualized in the scatter plots via mr_scatter_plotmr function; the effect size of potential exposure factors on sepsis was visualized via mr_forest_plot; and the symmetry of potential exposure factors distribution was displayed via the mr_funnel_plot function. Furthermore, the sensitivity analyses including heterogeneity test, pleiotropy test (P > 0.05), and leave-one-out (LOO) analysis were executed to evaluate the reliability of MR. The heterogeneity test was evaluated by the mr_heterogeneity function of “TwoSampleMR” package (v 0.6.4). If the results had heterogeneity (Q < 0.05), random effects IVW was adopted in the MR, otherwise fixed effects IVW would be adopted. The horizontal pleiotropy test was evaluated by the mr_pleiotropy_test function (Mr_presso function was used to perform an auxiliary test). The LOO analysis was executed via the mr_leaveoneout function, and the results were displayed via the mr_leaveoneout_plot function. Lastly, a Steiger test was conducted by the “TwoSampleMR” package (v 0.6.4) to verify whether the direction of the MR analysis was correct (correct causal direction = TRUE, Q < 0.05). Most importantly, the GM corresponding to IVs which were obtained through the above analysis were regard as key microorganisms for subsequent analysis. To control the risk of false positives arising from multiple testing, the Bonferroni correction method was applied to adjust the significance threshold. The adjusted significance threshold was set at P < 0.0083 (0.05/6).

### 2.3 Identification of metabolites and target of key microorganisms

The gutMGene database (http://bio-computing.hrbmu.edu.cn/gutmgene) was utilized to search metabolites corresponding to the key microorganisms at genus level, and the PubChem database (https://pubchem.ncbi.nlm.nih.gov/) was utilized to obtain simplified molecular input line entry system (SMILES) format for metabolites. Finally, the Similarity Ensemble Approach database (SEA) (https://sea.bkslab.org/) and Swiss Target Prediction database (STP) (http://www.swisstargetprediction.ch/) were utilized to acquired the targets genes, and the input file in the STP database was the SMILES of format for metabolites, After selecting all targets from 2 databases, the intersection genes were regarded as the targets of key microorganisms. Finally, a network was built via the Cytoscape software to import the correspondence between key microorganisms, metabolites, and target genes.

### 2.4 Identification of candidate genes

The “limma” package (v 3.56.2) (23) was utilized to acquire differentially expressed genes (DEGs) between SCM and control samples (SCM vs control) (|log_2_FoldChange (FC)| > 1, adj.P < 0.05) in all samples of GSE79962. Then, DEGs were visualized by the volcano plot utilizing the “ggplot2” package (v 3.5.1) (24), and the top 10 up or down-regulated genes labeled based on adj.P value. Moreover, the top 10 up or down regulated genes between the 2 groups were also displayed in a heat map via the “pheatmap” package (v 1.0.12) (25). Lastly, the candidate genes were obtained by intersecting the DEGs and the targets of key microorganisms with the “ggvenn” package (v 1.7.3).

### 2.5 Enrichment pathways of candidate genes

The Kyoto Encyclopedia of Genes and Genomes enrichment (KEGG), Gene Ontology (GO), and Disease Ontology (DO) analyses were applied via the “clusterProfiler” package (v 4.8.3) (26) to explore the functions of candidate genes and diseases linked to candidate genes (P < 0.05). The top 10 notably enriched terms in GO, KEGG, and DO were depicted based on the number of genes in terms. Besides, a network was constructed via Cytoscape software (v 3.10.2) to explore the relationship between key microorganisms, metabolites corresponding to the key microorganisms, KEGG pathways, and candidate genes. For enrichment analysis, GO P-values were adjusted using the Benjamini-Hochberg (BH) method for FDR correction, while KEGG and DO analyses also employed BH adjustment, with adjusted P < 0.05 considered significant.

### 2.6 Machine learning analysis

The support vector machine-recursive feature elimination (SVM-RFE), Random Forest (RF), and least absolute shrinkage and selection operator (LASSO) logistic regression analyses were conducted to obtain biomarkers for SCM. Firstly, the LASSO and SVM-RFE algorithms with 5 fold cross validation were conducted via the “glmnet” package (v 4.1-8) (27) and the “e1071” package (v 1.7-16) (28), respectively, in all samples of the GSE79962 dataset. At the same time, the RF algorithm was conducted via the “randomForest” package (v 4.7-1.2) (29) and the top 5 genes were selected. Afterwards, the biomarkers were obtained by the intersection of genes in 3 algorithms via the “ggvenn” package (v 1.7.3).

### 2.7 The expression levels and receiver operating characteristic analysis of biomarkers

Then, the expression differences of genes between SCM and control samples were evaluated via Wilcoxon test (P < 0.05) in the GSE79962 dataset. The receiver operating characteristic (ROC) curve of biomarkers was employed utilizing “pROC” package (v 1.18.0) (30) in the GSE79962 datasets to evaluate the recognition ability of biomarkers for SCM (area under curve (AUC) > 0.7).

### 2.8 Subcellular and chromosome localization of biomarkers

In order to explore the position of biomarkers on chromosomes, the “RCircos” package (v 1.2.2) (31) was used to visualize the position of biomarkers on chromosomes. In order to predict the location of biomarkers in cells, the GeneCards database (https://www.genecards.org/) was utilized to acquire subcellular localization of biomarkers, and the results were visualized via the “ggplot2” package (v 3.5.1).

### 2.9 Gene set enrichment analysis and GeneMANIA analysis

In order to explore the biological functions of biomarkers for SCM, the GSEA of each biomarker was performed in all samples of the GSE79962 dataset. Firstly, the “c2.cp.kegg.v2023.1.Hs.symbols.gmtt” gene set was utilized as the reference gene set, while the gene set was acquired from the Molecular Signatures Database (MsigDB) (https://www.gsea-msigdb.org/gsea/msigdb/). Then, the Spearman correlation between each biomarker and other genes was calculated by “psych” package (v 2.2.9), and the genes were sorted by their correlation coefficients in descending order subsequently. Lastly, the GSEA was executed via the “clusterProfiler” package (v 4.8.3) (|normalized enrichment score| (|NES|) > 1, P < 0.05), and the top 5 pathways were revealed based on the P value. To further investigate the interactions and functional associations between biomarkers and functionally similar genes, biomarkers were imported into the GeneMANIA database (http://genemania.org/) to construct an interaction network and selected the appropriate species as “Homo sapiens”. Additionally, GSEA P-values were adjusted using the BH method, with adjusted P < 0.05 and |NES| > 1 considered significant.

### 2.10 Immune infiltration analysis

The infiltration abundance of the 28 immune cells in all samples in GSE134347 was elucidated by the single-sample gene-set enrichment analysis (ssGSEA) algorithm of “GSVA” package (v 1.53.28) (32). Then, the differentially infiltrated cells (DICs) between SCM and control were obtained by Wilcoxon test (P < 0.05). After that, the correlations between biomarkers and DICs were calculated by Spearman analysis via the “psych” package (v 2.4.6.26) (|cor| > 0.3, P < 0.05). These results were displayed via the “ggplot2” package (v 3.5.1). The Wilcoxon rank-sum test was employed, and the BH method was applied for multiple comparison correction of P values (P value < 0.05). Correlation analysis was performed using Spearman’s method and was also subjected to BH multiple comparison correction.

### 2.11 Construction of molecular regulatory network

The transcription factors (TFs) that link to biomarkers were acquired in the Network Analyst database (https://www.networkanalyst.ca) (specify organism = “H. sapiens human”). The microRNAs (miRNAs) linked to biomarkers were predicted in the miRDB database (https://mirdb.org/) (target score > 80). The above results were visualized by Cytoscape (v 3.10.2).

### 2.12 Metabolites screening and molecular docking

The physical and chemical properties of metabolites linked to key microorganisms were calculated utilizing the SwissADME database (http://www.swissadme.ch/). Then, compounds with good oral absorption, distribution, metabolism, and excretion potential according to Lipinski’s five principles (molecular weight ≤ 500 g/mol, HBA ≤ 10, HBD ≤ 5, MlogP ≤ 5, TPSA ≤ 140, Lipinski’s violations ≤ 1, and bioavailability Score > 0.1) were reserved, and key metabolites were obtained. Then, the sores of 7 key safety indicators including hERG inhibition, human hepatotoxicity (H-HT), drug-induced liver injury (DILI), respiratory, rat oral acute, carcinogenicity and skinSen of key metabolites were obtained in the ADMETlab 3.0platform (https://admetlab3.scbdd.com/). The total score for each metabolite were added by these 7 scores. The lower the total score, the less toxic the metabolite is. To explore whether biomarkers could bind with the metabolites, molecular docking was performed based on metabolites with the lowest total score and biomarkers. Firstly, the UniProt database (https://www.uniprot.org/) was utilized to obtain the 3D molecular structure of encoded proteins for biomarkers, and the PubChem database (https://pubchem.ncbi.nlm.nih.gov/) was utilized to obtain the 3D molecular structure of metabolites. Molecular docking was conducted, and the interaction force analysis between metabolites and biomarkers was conducted by CB-DOCK database (https://cadd.labshare.cn/cb-dock/php/blinddock.php), and the results were visualized by the PyMOL software (score ≤ -1.2 kcal/mol).

### 2.13 Molecular dynamics simulation analysis

MD simulation was conducted via the GROMACS software (v 2024.4) (33) to further validate the results of molecular docking. Firstly, the TIP3P (TIP3 point) water model was utilized, and the shape of the box was set as a cube. At the same time, a distance of 1 nanometer between the edge of the box and the edge of the protein was ensured, and the ions were added to maintain the electrical neutrality of the entire system. The energy consumption of the entire system was reduced to the maximum extent possible using the steepest descent method. Then, pre balancing was carried out in 2 stages: an NVT system was utilized to simulate balancing at 300K and 100ps in the first stage; an NPT system was utilized to simulate balancing at a size was 2 fs and 100ps in the second stage. The 20–100 ns MD simulation was conducted. The MD simulation results, including RMSD (Root Mean Square Deviation), RMSF (Root Mean Square Fluctuation), Rg (Radius of Gyration), changes in total energy and number of hydrogen bonds.

### 2.14 Experimental validation

Blood samples were collected from three patient groups (sepsis group, sepsis cardiomyopathy group, and healthy control group) at the Xinjiang Uygur Autonomous Region People’s Hospital. All participants signed informed consent forms. This study was approved by the Ethics Committee of the Xinjiang Uygur Autonomous Region People’s Hospital. The detailed baseline clinical characteristics of the enrolled participants are summarized in **Supplementary Table 1**.

IL-6 and cTnI concentrations were measured in serum samples from the three groups using ELISA to assess inflammatory levels and myocardial injury. Test samples and standards were added to pre-coated plate wells, followed by the corresponding antibody mixture. The plate was sealed and incubated at room temperature with shaking for 1 hour. After incubation, wells were thoroughly washed with washing buffer. TMB chromogenic solution was added, and the mixture was shaken in the dark to develop color. The reaction was terminated by adding a stop solution. An ELISA reader measured the optical density (OD) values at 450 nm for each well. A standard curve was plotted based on the concentrations of standards and their corresponding OD values, and the concentrations of IL-6 and cTnI in the samples were calculated.

Propylene glycol concentrations in clinical samples from three groups were assessed using gas chromatography–mass spectrometry (GC-MS). A series of standard working solutions was prepared using commercial blank serum. Accurately measured serum samples were subjected to liquid-liquid extraction with dichloromethane. After vortex mixing, ultrasonication, and centrifugation, the supernatant was filtered to obtain the test solution for GC-MS analysis. Instrumental analysis employed programmed temperature conditions. Following mass spectrometry detection, propylene glycol concentrations in each sample were calculated based on the standard curve.

Reverse transcription quantitative PCR (RT-qPCR) was employed to assess biomarker expression in samples. Total RNA was extracted using TRIzol, followed by reverse transcription to obtain complementary DNA (cDNA). Quantitative PCR utilized cDNA as template with primers listed in **Supplementary Table 2**, and gene expression levels were measured using the 2^-ΔΔCt method.

Statistical analysis and visualization of the experimental results were performed using GraphPad Prism software.

### 2.15 Statistical analysis

Bioinformatics analyses were conducted via the R programming language(v 4.3.3). The Wilcoxon test was utilized to contrast the distinctions between 2 groups, and one-way analysis of variance (One-way ANOVA) was used to compare the overall differences among the three groups. If the differences were significant, then the Tukey *post-hoc* test was employed for pairwise comparisons. P < 0.05 was considered notably significant.

## 3 Results

### 3.1 Identification of key microorganisms

**Figure 1:**
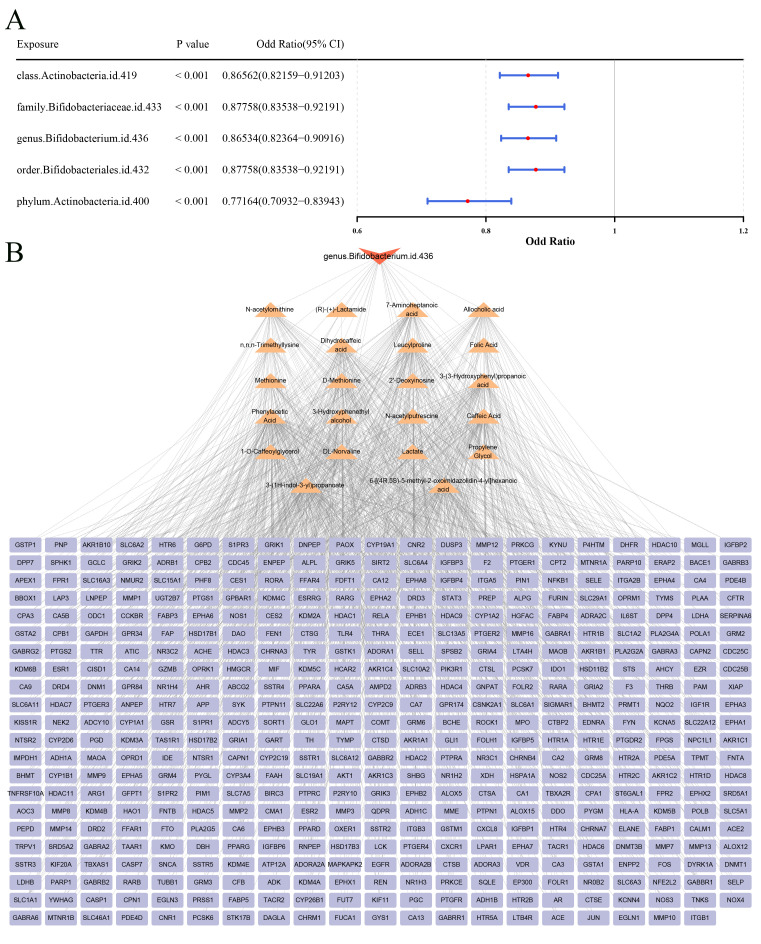
Mendelian randomization analysis of intestinal microbiota and acquisition of metabolites and related targets of key intestinal microbiota. **(A)** IVW forest map. **(B)** Key microbial-metabolism-target gene network diagram. Red nodes represent key microorganisms, yellow nodes represent metabolites predicted by key microorganisms, and blue nodes represent target genes predicted by metabolites.

In MR analysis, 5 GM that had significant causal relationships with sepsis were obtained based on IVW results (P < 0.05) (**Figure 1A**). The scatter plot indicated that the *class.Actinobacteria.id.419* (OR = 0.86562, 95%CI: 0.82159 - 0.91203), *family.Bifidobacteriaceae.id.433* (OR = 0.877588, 95%CI: 0.83538 - 0.92191), *genus.Bifidobacterium.id.436.* (OR = 0.86534, 95%CI: 0.82364 - 0.90916), *order.Bifidobacteriales.id.432* (OR = 0.87758, 95%CI: 0.83538 - 0.92191), and phylum.Actinobacteria.id.400 (OR = 0.77167, 95%CI: 0.70932 - 0.83943) were protective factors for sepsis (**Supplementary Figure 1**). In addition, the forest map showed the same results as the scatter plot (**Supplementary Figure 2**). The funnel plot revealed that a generally symmetrical and uniform distribution of SNPs in the GM group, indicating that the selected IVs conformed to Mendel’s second law (**Supplementary Figure 3**). In the heterogeneity test, the Q values of 5 GM were greater than 0.05, indicating that there was no heterogeneity (**Table 1**). Furthermore, the Q values of 5 GM were greater than 0.05 in the horizontal pleiotropy test, indicating that there was no horizontal pleiotropy in the results (**Table 2**). The results of LOO suggested that when each SNP was gradually removed, the remaining SNPs had little effect on the sepsis, indicating that the MR results were reliable (**Supplementary Figure 4**). The Steiger test results indicated that these 5 GM, including the *class.Actinobacteria.id.419*, *family.Bifidobacteriaceae.id.433*, *genus.Bifidobacterium.id.436.scatter*, *order.Bifidobacteriales.id.432.scatter* and *phylum.Actinobacteria.id.400*, had unilateral causal connections with sepsis (**Table 3**). After Bonferroni correction, the causal associations between these 5 gut microbiota taxa and sepsis remained statistically significant based on the inverse variance weighted method, confirming their robustness as key protective factors (adjusted P < 0.0083) (**Table 4**). Therefore, these 5 GM were considered as key microorganisms.

**Table 1:** The results of heterogeneity test.

id.exposure	id.outcome	Method	Q	Q_df	Q_pval
class.Actinobacteria.id.419	Sepsis||id:ieu-b-4980	MR Egger	31.1895066721807	46	0.953381711530856
		Inverse variance weighted	31.1899979666954	47	0.963253394393462
family.Bifidobacteriaceae.id.433		MR Egger	33.2552961996784	48	0.947841108956406
		Inverse variance weighted	33.255309142211	49	0.958435046839213
genus.Bifidobacterium.id.436		MR Egger	24.0514907430635	46	0.996874096983888
		Inverse variance weighted	24.4299607777461	47	0.997353534306518
order.Bifidobacteriales.id.432		MR Egger	33.2552961996784	48	0.947841108956406
		Inverse variance weighted	33.255309142211	49	0.958435046839213
phylum.Actinobacteria.id.400		MR Egger	2.84173364496536	22	0.999999673405536
		Inverse variance weighted	2.99232929924347	23	0.999999808989559

**Table 2:** The results of horizontal pleiotropy test.

id.exposure	id.outcome	egger_intercept	se	pval
class.Actinobacteria.id.419	Sepsis||id:ieu-b-4980	-0.000399301	0.0180147948620387	0.982412076173614
family.Bifidobacteriaceae.id.433		6.23479582077985e-05	0.0173305602422607	0.997144468524598
genus.Bifidobacterium.id.436		0.0104692717084873	0.0170176944295636	0.541454912696813
order.Bifidobacteriales.id.432		6.23479582077985e-05	0.0173305602422607	0.997144468524598
phylum.Actinobacteria.id.400		-0.019222567	0.049534200560243	0.701696392985782

**Table 3:** The results of steiger test.

id.exposure	id.outcome	snp_r2.exposure	snp_r2.outcome	correct_causal_direction	steiger_pval
class.Actinobacteria.id.419	Sepsis||id:ieu-b-4980	0.152116381842519	0.000134221778718495	TRUE	0
family.Bifidobacteriaceae.id.433		0.154209818294618	0.000133604501563714	TRUE	0
genus.Bifidobacterium.id.436		0.150326828601084	0.000127722366726309	TRUE	0
order.Bifidobacteriales.id.432		0.154209818294618	0.000133604501563714	TRUE	0
phylum.Actinobacteria.id.400		0.0590092901616078	8.09828653015343e-05	TRUE	2.79804700062243e-221

**Table 4:** Bonferroni-corrected causal estimates for gut microbiota taxa.

id.outcome	Outcome	Exposure	Method	nsnp	b	se	pval	or	bonf_p
Sepsis||id:ieu-b-4980	Sepsis	phylum.Actinobacteria.id.400	Inverse variance weighted	24	-0.259	0.043	1.603e-09	0. 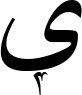	9.620e-09
Sepsis||id:ieu-b-4980		genus.Bifidobacterium.id.436		48	-0.145	0.025	9.514e-09	0.865	5.708e-08
Sepsis||id:ieu-b-4980		class.Actinobacteria.id.419		48	-0.144	0.027	6.073e-08	0.866	3.644e-07
Sepsis||id:ieu-b-4980		family.Bifidobacteriaceae.id.433		50	-0.131	0.0251	2.062e-07	0.878	1.237e-06
Sepsis||id:ieu-b-4980		order.Bifidobacteriales.id.432		50	-0.131	0.0251	2.062e-07	0.878	1.237e-06

### 3.2 Candidate genes linked to SCM and GM

**Figure 2:**
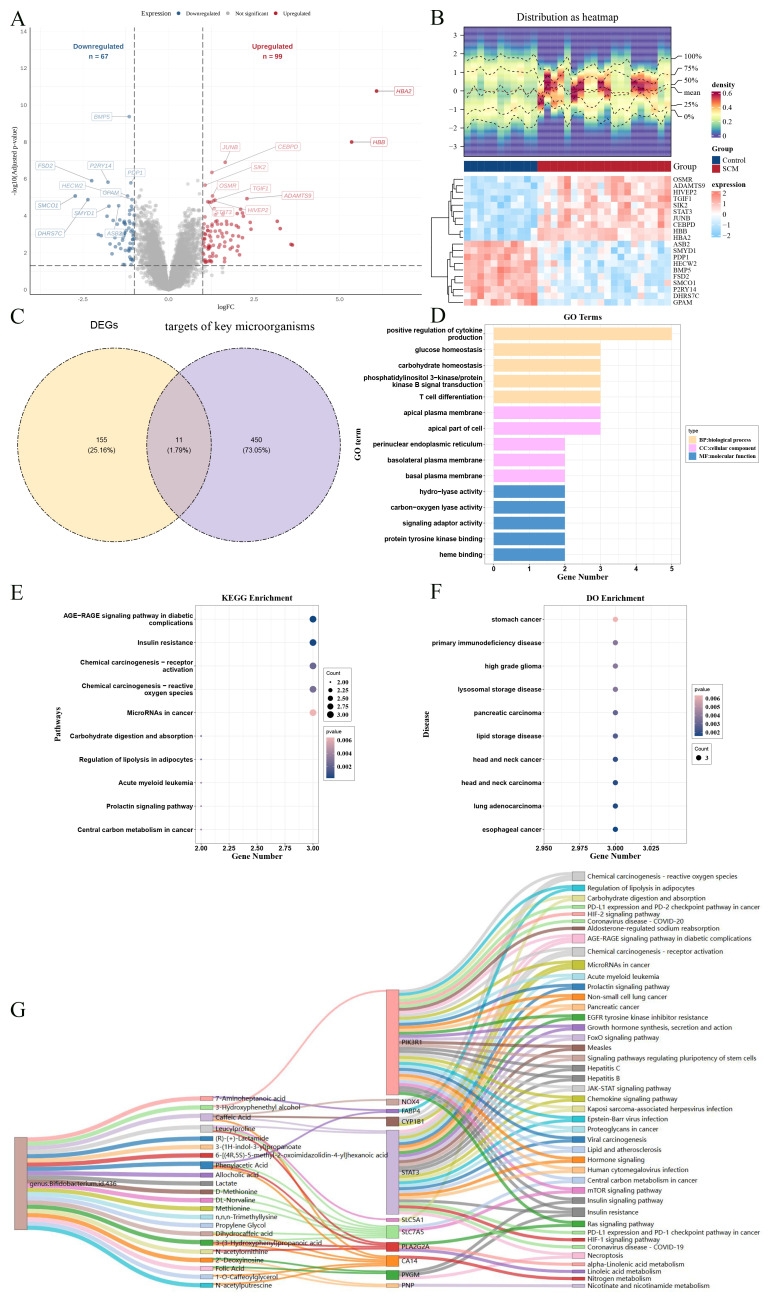
Identification of DEG, acquisition and enrich analysis of candidate genes. **(A)** Volcano plot for differential analysis of the GSE79962 dataset, highlighting significant variations in gene expression. Red dots represent upregulated genes, blue dots represent downregulated genes, and gray dots represent genes with no significant difference. **(B)** Expression density heatmap and expression heatmap. The expression density heatmap at the upper part of the figure shows kernel density estimation of expression distribution for each gene, with red colors indicating higher density. At the bottom of the figure is the expression heatmap. **(C)** Intersection Venn diagram of differentially expressed genes and key microbial targets. **(D)** Diagram of the GO analysis. **(E)** Diagram of the KEGG analysis. **(F)** Diagram of the DO analysis. **(G)** Sankey diagram of the microbial-metabolism-target-KEGG pathway.

In this study, the key microorganisms at the genus level only included *genus.Bifidobacterium.id.436.scatter*. So, a total of 22 metabolites such as folic acid and 461 targets such as CA12 of the *genus.Bifidobacterium.id.436.scatter* were obtained in the databases (**Figure 1B**; **Supplementary Table 3**). In the GSE79962 dataset, a total of 166 DEGs were determined, of which 99 genes were up-regulated in the SCM group (**Figures 2A, B**). Then, 11 candidate genes were acquired through the intersection of the targets of GM and SCM (**Figure 2C**). In GO analysis, a total of 434 biological processes (BPs), such as glucose homeostasis, 12 cellular components (CCs) such as perinuclear endoplasmic reticulum, and 73 molecular functions (MFs) such as signaling adaptor activity were enriched by candidate genes respectively (P < 0.05) (**Figure 2D**; **Supplementary Table 4**). In KEGG analysis, the candidate genes were enriched in 40 KEGG pathways such as chemical carcinogenesis - receptor activation (**Figure 2E**; **Supplementary Table 5**) (P < 0.05). In DO analysis, the candidate genes were linked to 94 diseases such as primary immunodeficiency disease (**Figure 2F**; **Supplementary Table 6**) (P < 0.05). The network indicated 22 metabolites such as folic acid, and 21 KEGG pathways such as mTOR signaling pathway were linked to 11 candidate genes (**Figure 2G**). After multiple comparison correction using the BH method, enrichment analysis of candidate genes identified 75 GO terms, 13 KEGG pathways, and 21 DO terms. The majority of enriched terms remained consistent before and after correction, indicating that the key functional associations were not driven by statistical inflation (**Supplementary Figures 5A–C**).

### 3.3 Identification of biomarkers

**Figure 3:**
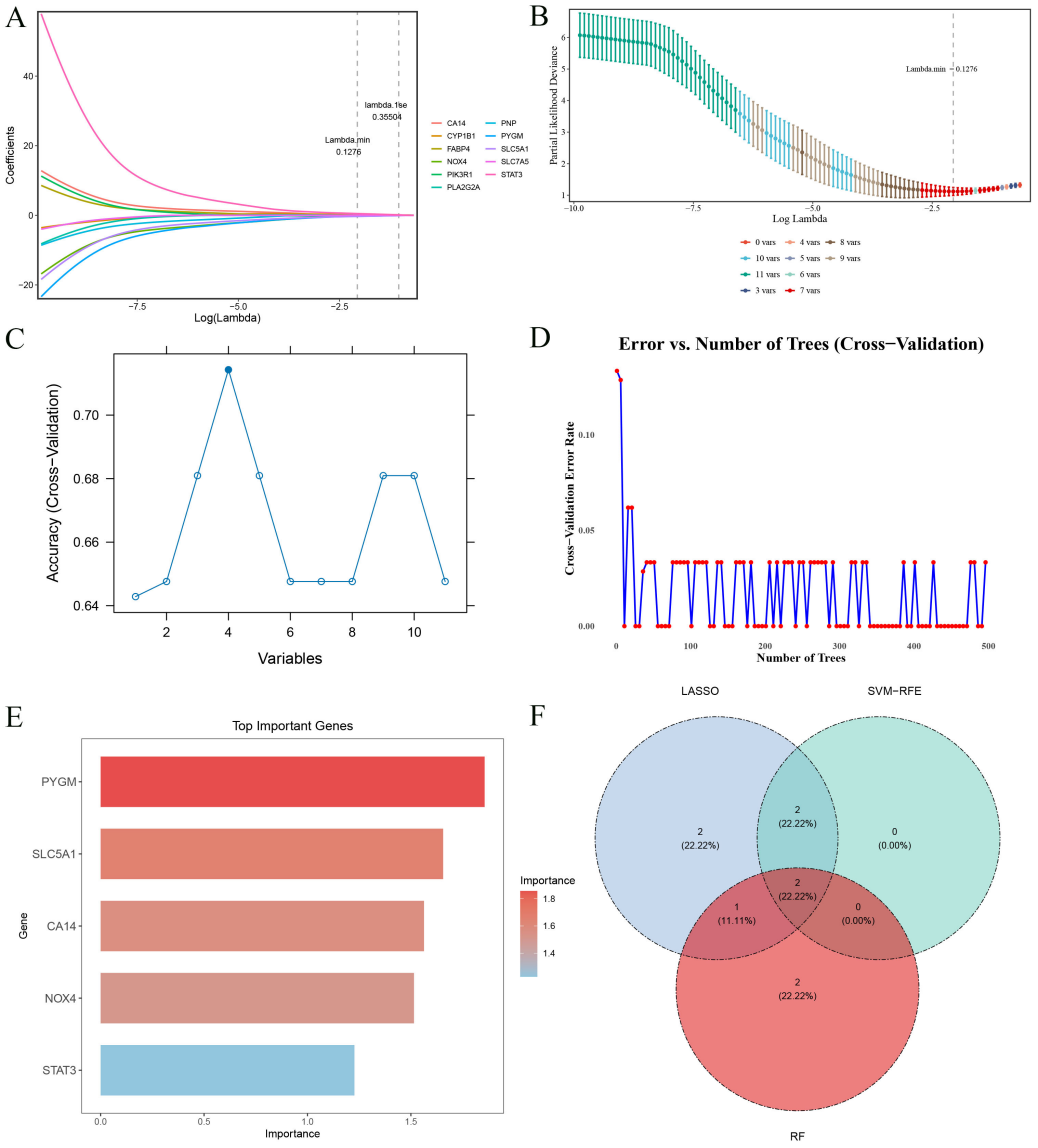
Machine learning screening. **(A)** Spectrum of LASSO coefficients. **(B)** The process of selecting the optimal parameter l for the LASSO regression model using cross-validation. **(C)** Results of the SVM-RFE algorithm. **(D)** Error rate curves for random forest models. **(E)** Feature importance ranking graph of RF. The redder the color, the higher the score. **(F)** Venn diagram of genes investigated by LASSO, SVM and RF.

**Figure 4:**
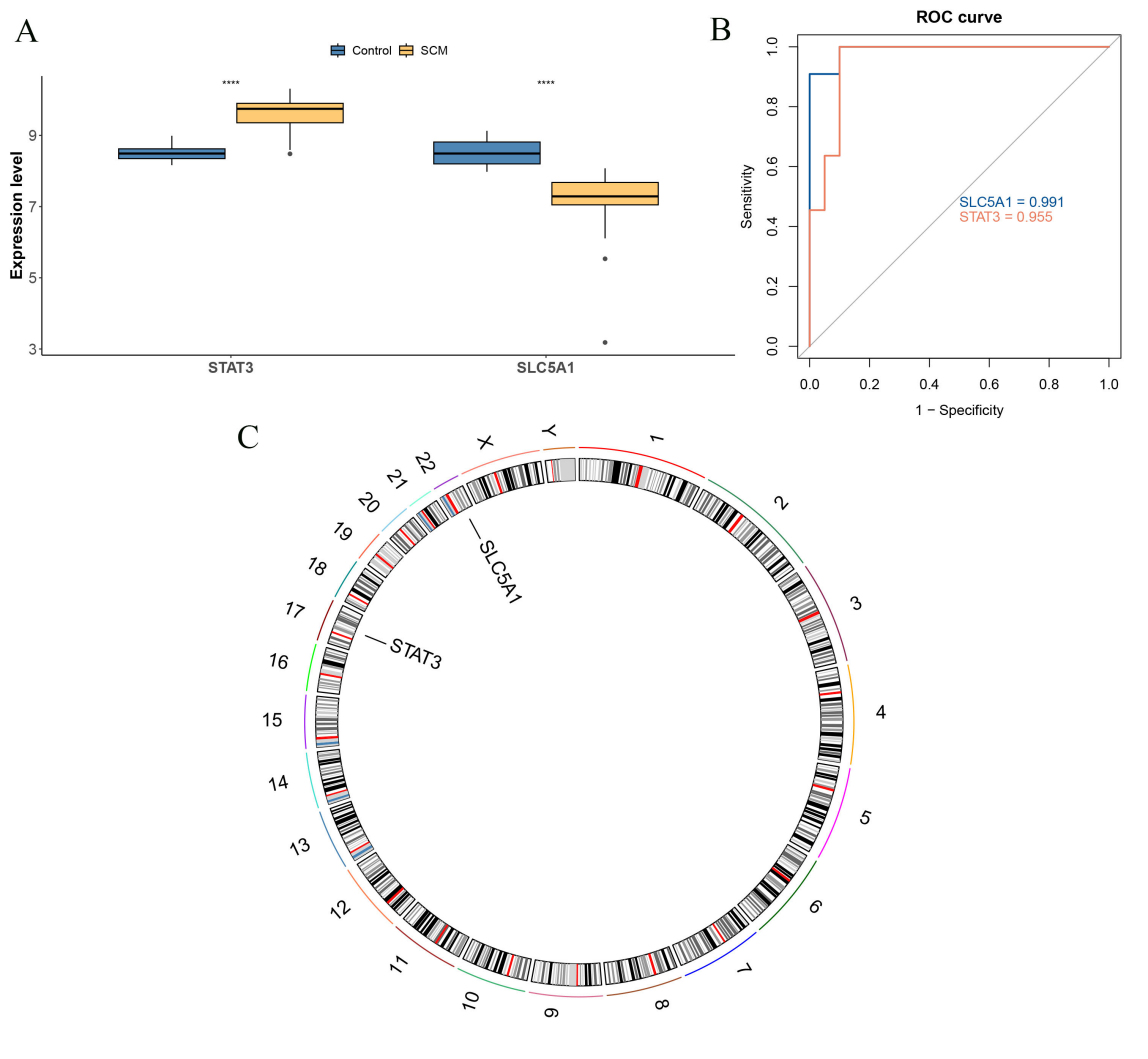
Expression levels, ROC curve analysis and chromosomal localization analysis of key biomarkers. **(A)** Expression analysis of key biomarkers. ****, p<0.0001. **(B)** ROC curve analysis of key biomarkers. **(C)** Chromosomal mapping circles of key biomarkers.

A total of 7 genes (STAT3, SLC7A5, PLA2G2A, CYP1B1, PYGM, PIK3R1, and SLC5A1) were obtained utilizing the LASSO algorithm (lambda.min = 0.1276) (**Figures 3A, B**), and 4 genes were selected in the SVM-RFE algorithm (SLC5A1, CYP1B1, STAT3, and PIK3R1) (**Figure 3C**), and 5 genes were selected utilizing the RF algorithm (PYGM, SLC5A1, CA14, NOX4, and STAT3) (**Figures 3D, E**). Then, 2 biomarkers (STAT3 and SLC5A1) were obtained from the intersection gene set of the 3 algorithms (**Figure 3F**). Finally, STAT3 and SLC5A1 showed notable differences between SCM and control samples (P < 0.05). STAT3 showed upward trend while SLC5A1 showed downward trend in the SCM group (**Figure 4A**). Then, the AUC of STAT3 (AUC = 0.955) and SLC5A1 (AUC = 0.991) was greater than 0.7, indicating these 2 biomarkers could effectively distinguish SCM and control samples (**Figure 4B**). The chromosome localization results indicated that STAT3 was localized on chromosome 17 and SLC5A1 on chromosome 22 (**Figure 4C**).

### 3.4 Enrichment pathway of biomarkers and DICs linked to biomarkers

**Figure 5:**
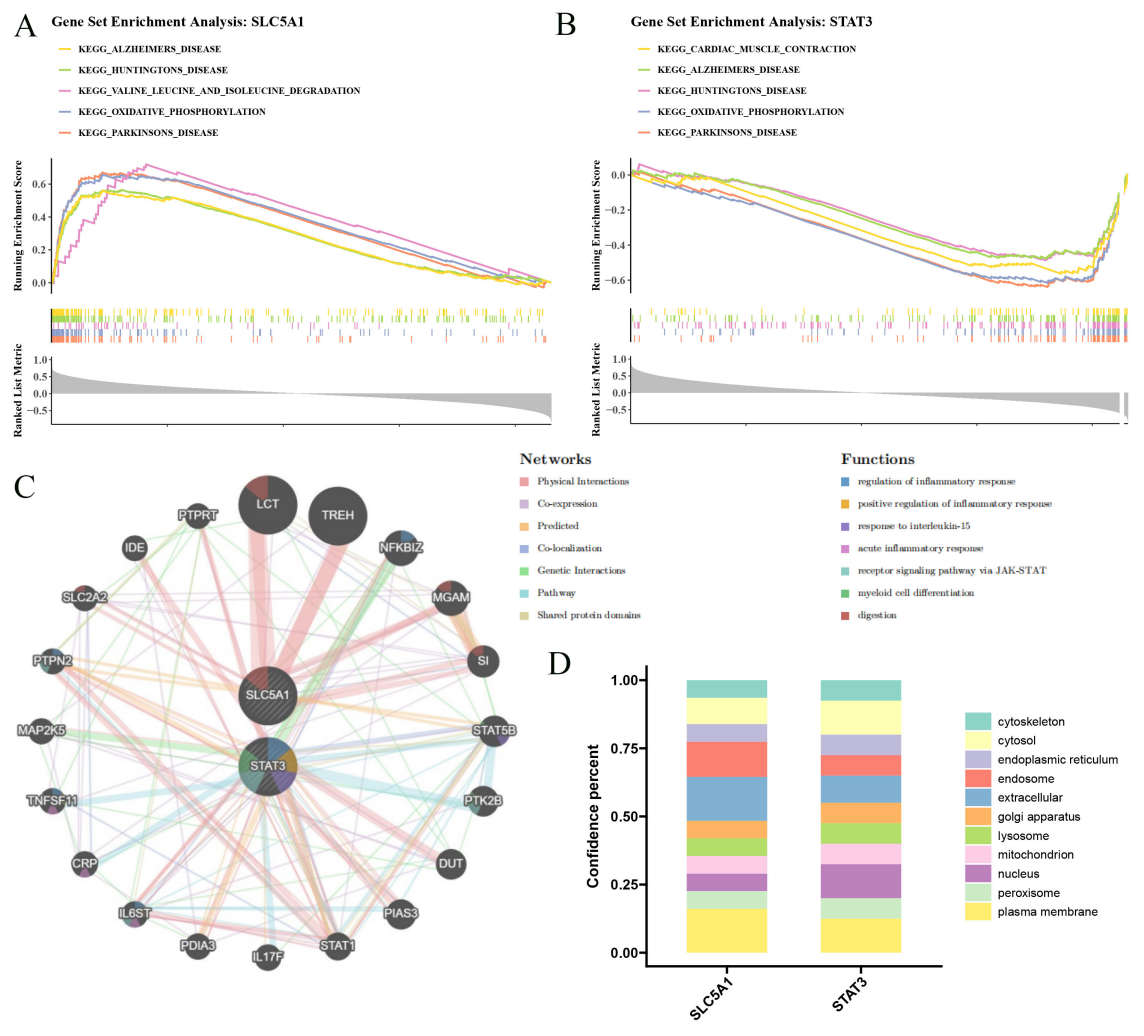
GSEA analysis, GeneMANIA analysis and subcellular localization analysis of key biomarkers. **(A)** GSEA result map of SLC5A1. The region to the left of 0 represents genes positively correlated with the key biomarkers, while the region to the right of 0 represents genes negatively correlated with the key biomarkers. Lines in different colors represent different biological pathways, and the small vertical lines in different colors below the lines represent individual genes enriched in the corresponding pathways. **(B)** GSEA result map of STAT3. **(C)** GeneMANIA analysis of key biomarkers. **(D)** Bar graph of cellular localization of key biomarkers.

**Figure 6:**
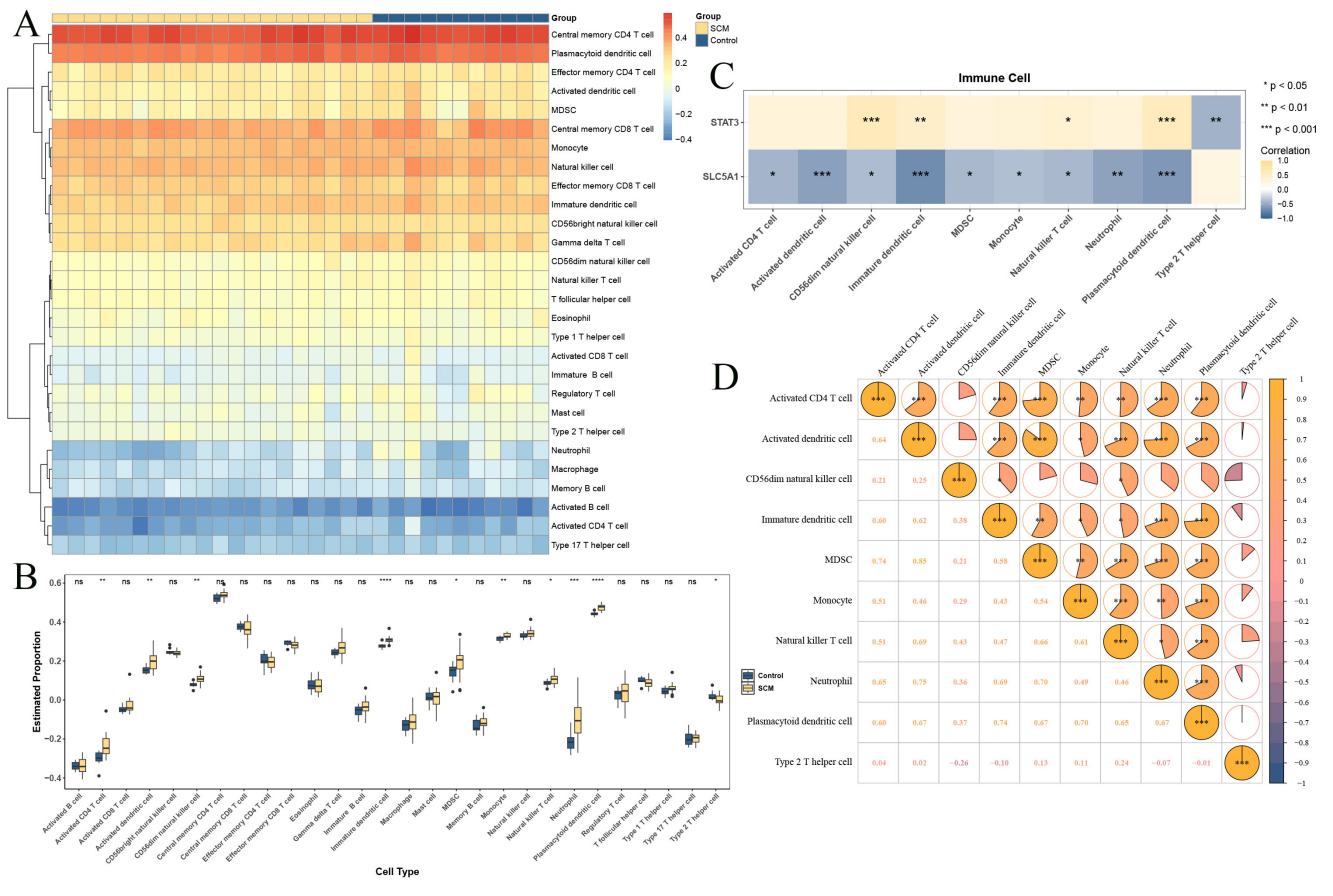
Immune infiltration analysis of key biomarkers. **(A)** Heat map of relative abundance of immune cells. The colored grid indicates the cellular content of the cells in the sample. **(B)** Box plot of immune cell infiltration abundance. **(C)** Heat map of correlation between key biomarkers and differential immune cells. **(D)** Correlation analysis between differential immune cells. ns, not significant; *p<0.05; **p<0.01; ***p<0.001; ****p<0.0001.

The GSEA results showed that the numbers of notable pathways enriched by STAT3 and SLC5A1 were 53 and 59, respectively (|NES| > 1, P < 0.05) (**Supplementary Table 7**). For example, MCM6 and NR3C1 were both enriched in valine leucine and isoleucine degradation, etc, indicating that biomarkers most likely affected the development of SCM through these notably enriched pathways (**Figures 5A, B**; **Supplementary Table 7**). After multiple comparison correction using the BH method, the top 5 significantly enriched pathways identified by GSEA for STAT3 and SLC5A1 remained entirely consistent with those obtained before correction (**Supplementary Figures 5D, E**). As shown in **Figure 5C**, the top 20 genes such as LCT had the strongest functional correlation to biomarker and the function which was enriched by these genes included regulation of inflammatory response. The subcellular localization results indicated that STAT3 was localized on plasma membrane and nucleus, SLC5A1 was localized on plasma membrane and extracellular (**Figure 5D**), indicating that biomarkers might play their main roles in plasma membrane. The abundance of 28 types of immune cells between SCM and control samples was displayed in **Figure 6A**. Then, 11 DICs such as naive B cells were obtained (P < 0.05) (**Figure 6B**). Then, SLC5A1 was notably negatively correlated with 10 DICs while STAT3 was notably positively correlated with 5 DICs (**Figure 6C**), and correlation results demonstrated that MDSCs was notably positively correlated with activated dendritic cells (cor = 0.85, P < 0.05) (**Figure 6D**). These results showed that these DICs such as MDSCs might have a notable impact on biomarkers on SCM. After BH correction, 7 of the 10 originally identified differential immune cells remained significantly different, and the correlation analysis results were largely consistent with the original findings, indicating the robustness of the identified immune infiltration differences and the associations between key genes and immune cells (**Supplementary Figures 5F–I**).

### 3.5 Upstream factors linked to biomarkers

**Figure 7:**
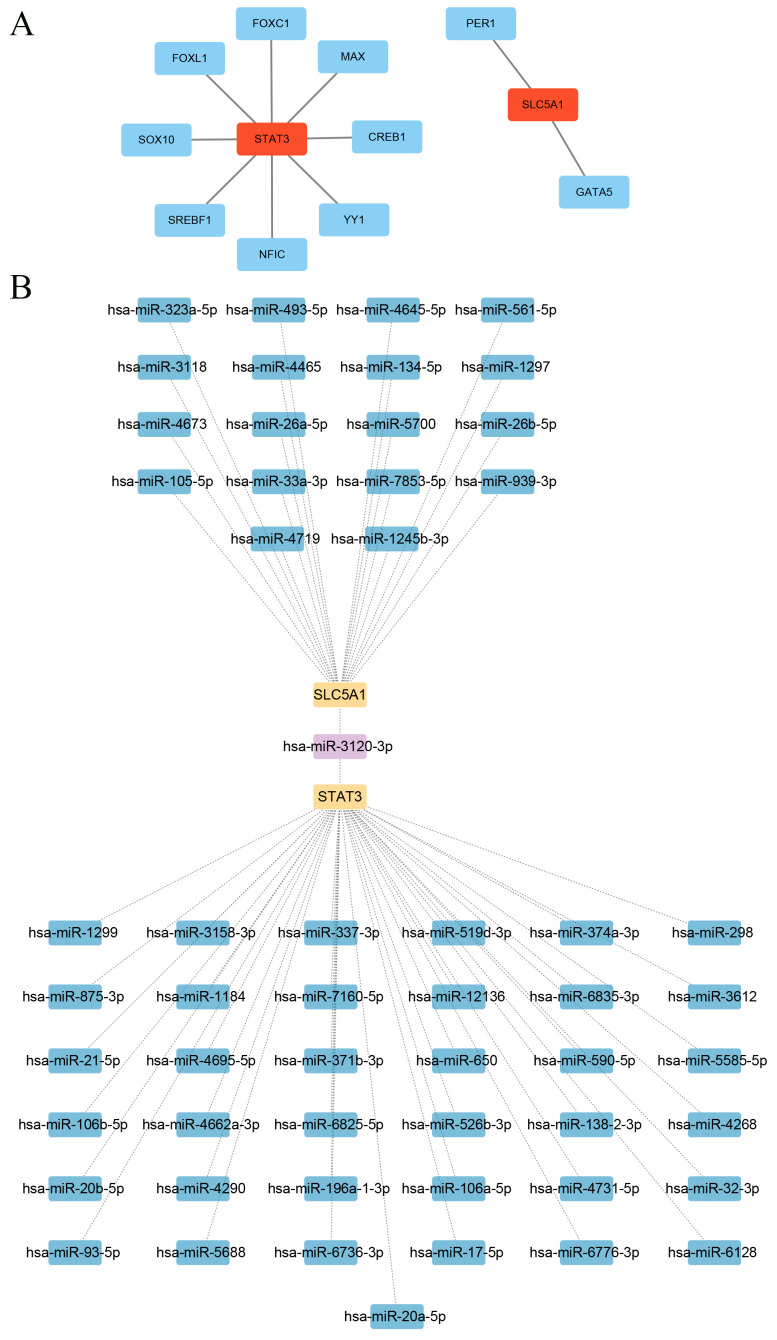
Molecular regulatory network construction. **(A)** Diagram of the mRNA-TF network of key biomarkers. Red nodes represent key biomarkers and blue nodes represent transcription factors. **(B)** Diagram of the key biomarker mRNA-miRNA network. Yellow nodes represent key biomarkers, blue nodes represent miRNAs, and purple nodes are miRNAs shared by key biomarkers.

A total of 8 TFs such as FOXC1 were found to be associated with STAT3, and 2 TFs such as PER1 were found to be associated with SLC5A1 (**Figure 7A**). Moreover, a total of 38 miRNAs such as hsa-miR-519d-3P and hsa-miR-6835-3p were associated with STAT3, and 18 miRNAs such as hsa-miR-5700 were associated with SLC5A1 (**Figure 7B**). Therefore, these factors linked to biomarkers might have important roles in SCM progression.

### 3.6 The metabolites link to biomarkers

**Figure 8:**
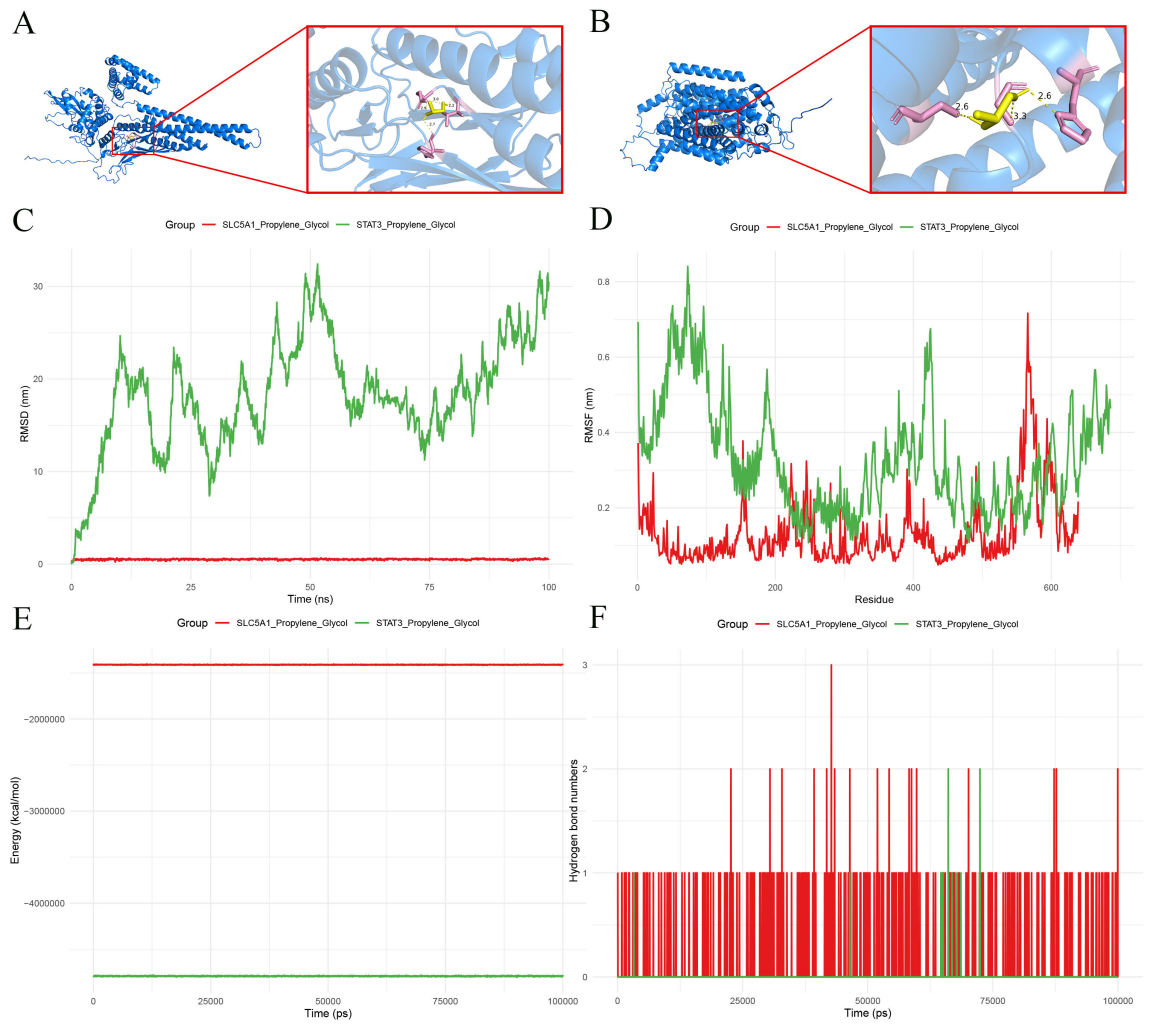
Drug similarity and toxicity analysis of metabolites. **(A, B)** Molecular docking results of STAT3-Propylene Glycol and SLC5A1-Propylene Glycol. The blue helix structure is the receptor protein, the yellow stick model is the active molecule Propylene Glycol, the nearby yellow dashed line is the hydrogen bond formed between the active ingredient and the amino acid residues, the pink stick is the amino acid bound to the active protein, the number under the dashed line indicates the hydrogen bond length, and the English character indicates the name of the amino acid residues. **(C)** Changes in RMSD from molecular dynamics simulations. **(D)** Changes in RMSF from molecular dynamics simulations. **(E)** Energy variation of molecular dynamics simulations. **(F)** Hydrogen bond changes from molecular dynamics simulations.

In the Pubchem database, SMILES of 21 metabolites were obtained while 2’-Deoxyinosine was removed because the SMILES could not be found (**Table 5**). Then, 20 metabolites were reserved in the SwissADME database while folic Acid violated Lipinski’s rule (HBD = 6, TPSA = 213.28), was excluded (**Table 6**). After that, the toxicological evaluation of metabolites was shown in **Table 7** and propylene glycol was selected to perform molecular docking because the total score was the lowest. The molecular docking results showed that propylene glycol had a good binding mode with STAT3 (score = -3.7 kcal/mol) (**Figure 8A**; **Table 8**) and SLC5A1 (score = -3.7 kcal/mol) (**Figure 8B**; **Table 8**). These results indicated that propylene glycol might represent a candidate metabolite associated with SCM pathology. The RMSD results revealed the binding stability of STAT3 and propylene glycol was higher than that of SLC5A1 and Propylene Glycol (**Figure 8C**). The RMSF results revealed the flexibility of STAT3 and Propylene Glycol, as well as SLC5A1 and Propylene Glycol was good (**Figure 8D**), and the total energy remained stable during simulation time (**Figure 8E**). In addition, less variation in the number of hydrogen bonds of STAT3 was occurred during the interaction between small molecules and proteins, while that of SLC5A1 was opposite (**Figure 8F**). The results also indicated that propylene glycol had good binding activity with biomarkers.

**Table 5:** SMILES of 21 candidate metabolites.

Metabolite	SMILES
(R)-(+)-Lactamide	C[C@H](C(=O)N)O
1-O-Caffeoylglycerol	C1=CC(=C(C=C1/C=C/C(=O)OCC(CO)O)O)O
2’-Deoxyinosine	NA
3-(1H-indol-3-yl)propanoate	C1=CC=C2C(=C1)C(=CN2)CCC(=O)[O-]
3-(3-Hydroxyphenyl)propanoic acid	C1=CC(=CC(=C1)O)CCC(=O)O
3-Hydroxyphenethyl alcohol	C1=CC(=CC(=C1)O)CCO
6-[(4R, 5S)-5-methyl-2-oxoimidazolidin-4-yl]hexanoic acid	C[C@H]1[C@H](NC(=O)N1)CCCCCC(=O)O
7-Aminoheptanoic acid	C(CCCN)CCC(=O)O
Allocholic acid	C[C@H](CCC(=O)O)[C@H]1CC[C@@H]2[C@@]1([C@H](C[C@H]3[C@H]2[C@@H](C[C@@H]4[C@@]3(CC[C@H](C4)O)C)O)O)C
Caffeic Acid	C1=CC(=C(C=C1/C=C/C(=O)O)O)O
D-Methionine	CSCC[C@H](C(=O)O)N
Dihydrocaffeic acid	C1=CC(=C(C=C1CCC(=O)O)O)O
DL-Norvaline	CCCC(C(=O)O)N
Folic Acid	C1=CC(=CC=C1C(=O)N[C@@H](CCC(=O)O)C(=O)O)NCC2=CN=C3C(=N2)C(=O)NC(=N3)N
Lactate	CC(C(=O)[O-])O
Leucylproline	CC(C)C[C@@H](C(=O)N1CCC[C@H]1C(=O)O)N
Methionine	CSCC[C@@H](C(=O)O)N
N-acetylornithine	CC(=O)N[C@@H](CCCN)C(=O)O
N-acetylputrescine	CC(=O)NCCCCN
n, n, n-Trimethyllysine	C[N+](C)(C)CCCCC(C(=O)O)N
Phenylacetic Acid	C1=CC=C(C=C1)CC(=O)O
Propylene Glycol	CC(CO)O

**Table 6:** Physical and chemical properties of 21 metabolites.

Molecule	Canonical SMILES	Lipinski rule				Lipinski’s violations ≤ 1	Bioavailability score>0.1	TPSA<140A^2^
		MW ≤ 500	HBA ≤ 10	HBD ≤ 5	MLOGP ≤ 5			
(R)-(+)-Lactamide	C[C@H](C(=O)N)O	89.09	2	2	-1.25	0	0.55	63.32
1-O-Caffeoylglycerol	OCC(COC(=O)/C=C/c1ccc(c(c1)O)O)O	254.24	6	4	-0.07	0	0.55	107.22
3-(1H-indol-3-yl)propanoate	[O-]C(=O)CCc1c[nH]c2c1cccc2	188.2	2	1	1.4	0	0.85	55.92
3-(3-Hydroxyphenyl)propanoic acid	OC(=O)CCc1cccc(c1)O	166.17	3	2	1.37	0	0.85	57.53
3-Hydroxyphenethyl alcohol	OCCc1cccc(c1)O	138.16	2	2	1.21	0	0.55	40.46
6-[(4R, 5S)-5-methyl-2-oxoimidazolidin-4-yl]hexanoic acid	OC(=O)CCCCC[C@H]1NC(=O)N[C@H]1C	214.26	3	3	0.74	0	0.56	78.43
7-Aminoheptanoic acid	NCCCCCCC(=O)O	145.2	3	2	0.75	0	0.55	63.32
Allocholic acid	O[C@@H]1CC[C@]2([C@H](C1)C[C@H]([C@@H]1[C@@H]2C[C@H](O)[C@]2([C@H]1CC[C@@H]2[C@@H](CCC(=O)O)C)C)O)C	408.57	5	4	3.05	0	0.56	97.99
Caffeic Acid	OC(=O)/C=C/c1ccc(c(c1)O)O	180.16	4	3	0.7	0	0.56	77.76
D-Methionine	CSCC[C@H](C(=O)O)N	149.21	3	2	-2.2	0	0.55	88.62
Dihydrocaffeic acid	OC(=O)CCc1ccc(c(c1)O)O	182.17	4	3	0.79	0	0.56	77.76
DL-Norvaline	CCCC(C(=O)O)N	117.15	3	2	-2.2	0	0.55	63.32
Folic Acid	OC(=O)CC[C@@H](C(=O)O)NC(=O)c1ccc(cc1)NCc1cnc2c(n1)c(=O)[nH]c(n2)N	441.4	9	6	-0.62	2	0.11	213.28
Lactate	[O-]C(=O)C(O)C	89.07	3	1	-0.85	0	0.85	60.36
Leucylproline	CC(C[C@@H](C(=O)N1CCC[C@H]1C(=O)O)N)C	228.29	4	2	0.22	0	0.55	83.63
Methionine	CSCC[C@@H](C(=O)O)N	149.21	3	2	-2.2	0	0.55	88.62
N-acetylornithine	CC(=O)N[C@H](C(=O)O)CCCN	174.2	4	3	-0.64	0	0.55	92.42
N-acetylputrescine	NCCCCNC(=O)C	130.19	2	2	-0.01	0	0.55	55.12
n, n, n-Trimethyllysine	OC(=O)C(CCCC[N+](C)(C)C)N	189.28	3	2	-5.35	0	0.55	63.32
Phenylacetic Acid	OC(=O)Cc1ccccc1	136.15	2	1	1.66	0	0.85	37.3
Propylene Glycol	OCC(O)C	76.09	2	2	-0.63	0	0.55	40.46
(R)-(+)-Lactamide	C[C@H](C(=O)N)O	89.09	2	2	-1.25	0	0.55	63.32
1-O-Caffeoylglycerol	OCC(COC(=O)/C=C/c1ccc(c(c1)O)O)O	254.24	6	4	-0.07	0	0.55	107.22
3-(1H-indol-3-yl)propanoate	[O-]C(=O)CCc1c[nH]c2c1cccc2	188.2	2	1	1.4	0	0.85	55.92
3-(3-Hydroxyphenyl)propanoic acid	OC(=O)CCc1cccc(c1)O	166.17	3	2	1.37	0	0.85	57.53
3-Hydroxyphenethyl alcohol	OCCc1cccc(c1)O	138.16	2	2	1.21	0	0.55	40.46
6-[(4R, 5S)-5-methyl-2-oxoimidazolidin-4-yl]hexanoic acid	OC(=O)CCCCC[C@H]1NC(=O)N[C@H]1C	214.26	3	3	0.74	0	0.56	78.43
7-Aminoheptanoic acid	NCCCCCCC(=O)O	145.2	3	2	0.75	0	0.55	63.32
Allocholic acid	O[C@@H]1CC[C@]2([C@H](C1)C[C@H]([C@@H]1[C@@H]2C[C@H](O)[C@]2([C@H]1CC[C@@H]2[C@@H](CCC(=O)O)C)C)O)C	408.57	5	4	3.05	0	0.56	97.99
Caffeic Acid	OC(=O)/C=C/c1ccc(c(c1)O)O	180.16	4	3	0.7	0	0.56	77.76
D-Methionine	CSCC[C@H](C(=O)O)N	149.21	3	2	-2.2	0	0.55	88.62
Dihydrocaffeic acid	OC(=O)CCc1ccc(c(c1)O)O	182.17	4	3	0.79	0	0.56	77.76
DL-Norvaline	CCCC(C(=O)O)N	117.15	3	2	-2.2	0	0.55	63.32
Folic Acid	OC(=O)CC[C@@H](C(=O)O)NC(=O)c1ccc(cc1)NCc1cnc2c(n1)c(=O)[nH]c(n2)N	441.4	9	6	-0.62	2	0.11	213.28
Lactate	[O-]C(=O)C(O)C	89.07	3	1	-0.85	0	0.85	60.36
Leucylproline	CC(C[C@@H](C(=O)N1CCC[C@H]1C(=O)O)N)C	228.29	4	2	0.22	0	0.55	83.63
Methionine	CSCC[C@@H](C(=O)O)N	149.21	3	2	-2.2	0	0.55	88.62
N-acetylornithine	CC(=O)N[C@H](C(=O)O)CCCN	174.2	4	3	-0.64	0	0.55	92.42
N-acetylputrescine	NCCCCNC(=O)C	130.19	2	2	-0.01	0	0.55	55.12
n, n, n-Trimethyllysine	OC(=O)C(CCCC[N+](C)(C)C)N	189.28	3	2	-5.35	0	0.55	63.32
Phenylacetic Acid	OC(=O)Cc1ccccc1	136.15	2	1	1.66	0	0.85	37.3
Propylene Glycol	OCC(O)C	76.09	2	2	-0.63	0	0.55	40.46

**Table 7:** Toxicological indicators of 20 metabolites.

Metabolite	smiles	hERG	H-HT	Respiratory	Rat oral acute	Carcinogenicity	DILI	SkinSen	Total_score
Allocholic acid	C[C@H](CCC(=O)O)[C@H]1CC[C@H]2[C@@H]3[C@H](O)C[C@H]4C[C@H](O)CC[C@]4(C)[C@H]3C[C@H](O)[C@]12C	0.03913	0.51713	0.93381	0.51737	0.73030	0.21134	0.98705	3.936129961
Leucylproline	CC(C)C[C@H](N)C(=O)N1CCC[C@H]1C(=O)O	0.06670	0.90071	0.65346	0.36509	0.34596	0.65387	0.93682	3.922615789
Phenylacetic Acid	O=C(O)Cc1ccccc1	0.03026	0.36950	0.37734	0.36916	0.19715	0.91130	0.89729	3.152010405
Caffeic Acid	O=C(O)/C=C/c1ccc(O)c(O)c1	0.03780	0.68286	0.45941	0.07925	0.17864	0.69036	0.97550	3.103814974
n, n, n-Trimethyllysine	C[N+](C)(C)CCCCC(N)C(=O)O	0.11634	0.01036	0.98737	0.79665	0.16464	0.00003	0.94746	3.022855558
N-acetylputrescine	CC(=O)NCCCCN	0.06831	0.73957	0.38533	0.21270	0.35210	0.11198	0.95139	2.821395956
7-Aminoheptanoic acid	NCCCCCCC(=O)O	0.05063	0.45901	0.80436	0.23548	0.39189	0.08756	0.78859	2.817520987
3-(1H-indol-3-yl)propanoate	O=C(O)CCc1c[nH]c2ccccc12	0.03276	0.52040	0.67508	0.30118	0.45410	0.47142	0.24048	2.695411392
Methionine	CSCC[C@H](N)C(=O)O	0.03835	0.18962	0.12571	0.49391	0.71787	0.02847	0.95569	2.5496186
Dihydrocaffeic acid	O=C(O)CCc1ccc(O)c(O)c1	0.02811	0.42311	0.40999	0.19033	0.29598	0.29699	0.90448	2.548979431
3-Hydroxyphenethyl alcohol	OCCc1cccc(O)c1	0.12790	0.45759	0.29835	0.12590	0.46968	0.07026	0.91991	2.469583802
N-acetylornithine	CC(=O)N[C@@H](CCCN)C(=O)O	0.02533	0.60076	0.33300	0.20955	0.23533	0.11348	0.93340	2.450856322
DL-Norvaline	CCCC(N)C(=O)O	0.06719	0.27179	0.57919	0.45654	0.30410	0.02383	0.60301	2.305655105
(R)-(+)-Lactamide	C[C@@H](O)C(N)=O	0.02412	0.31112	0.36558	0.14861	0.46195	0.20747	0.72622	2.245079346
3-(3-Hydroxyphenyl)propanoic acid	O=C(O)CCc1cccc(O)c1	0.03396	0.46627	0.47729	0.20170	0.34330	0.17472	0.52634	2.223597437
6-[(4R, 5S)-5-methyl-2-oxoimidazolidin-4-yl]hexanoic acid	C[C@@H]1NC(=O)N[C@@H]1CCCCCC(=O)O	0.00948	0.33070	0.60643	0.17810	0.37392	0.10906	0.51514	2.122831668
D-Methionine	CSCC[C@@H](N)C(=O)O	0.03022	0.08960	0.06717	0.37633	0.42782	0.00774	0.99068	1.989552996
1-O-Caffeoylglycerol	O=C(/C=C/c1ccc(O)c(O)c1)OCC(O)CO	0.04847	0.32969	0.02395	0.03858	0.33627	0.11619	0.99767	1.890821587
Lactate	CC(O)C(=O)O	0.02783	0.33353	0.41564	0.27172	0.14892	0.17786	0.30437	1.679872392
Propylene Glycol	CC(O)CO	0.05225	0.30761	0.08616	0.07951	0.27379	0.02483	0.22517	1.049331402

**Table 8:** The results of molecular docking.

Biomarkers	Metabolites	Score
STAT3	Propylene Glycol	-3.7 kcal/mol
SLC5A1	Propylene Glycol	-3.7kcal/mol

### 3.7 Experimental validation

A total of 27 whole blood samples were included (control group: sepsis group: sepsis-associated cardiomyopathy group = 9:9:9). Differences in inflammatory markers, myocardial injury indicators, key metabolites, and their target gene expression levels across the three groups were preliminarily validated using ELISA, GC-MS, and RT-qPCR techniques.

**Figure 9:**
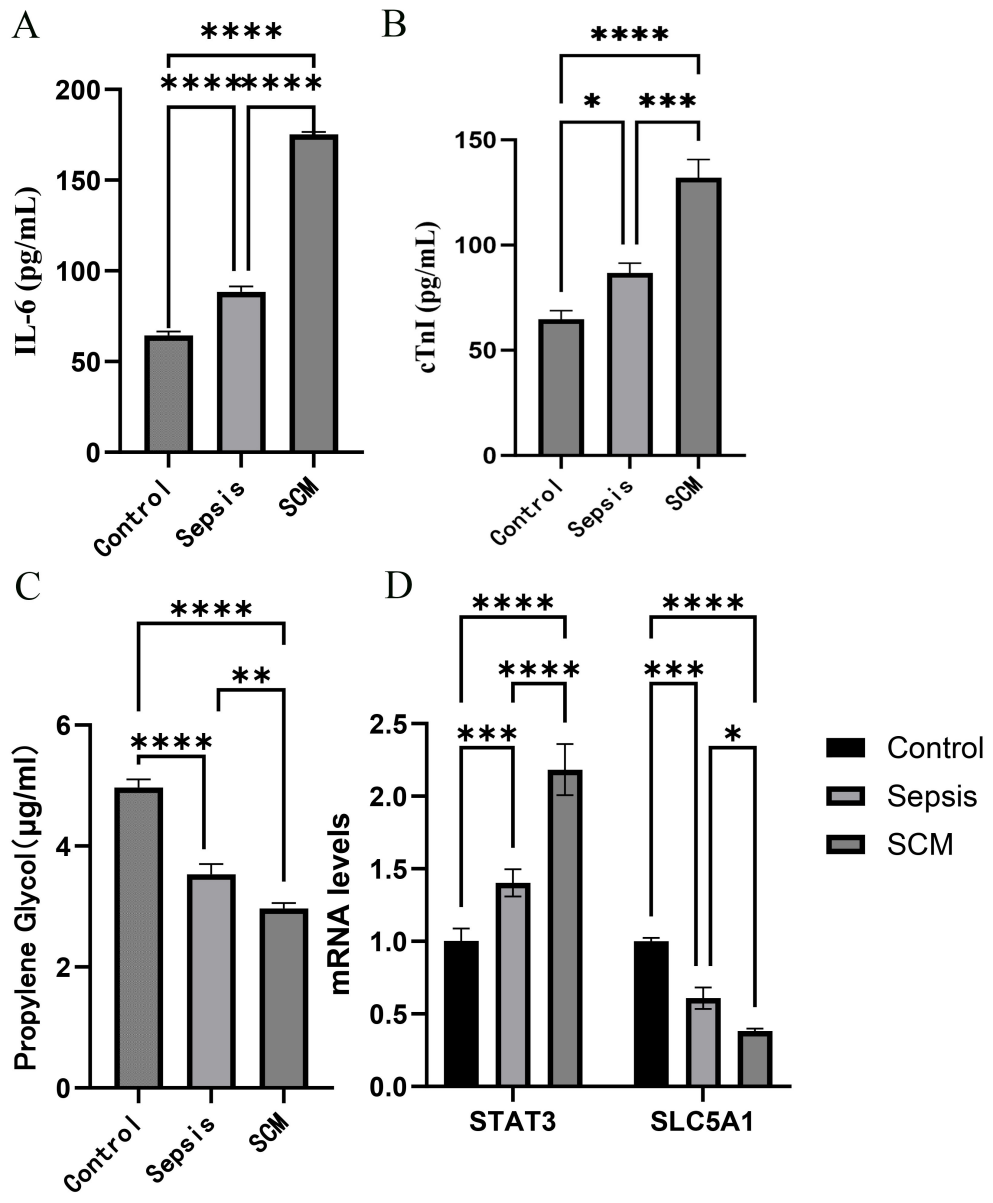
Clinical sample experimental validation. **(A)** Serum IL-6 concentration measured by ELISA. **(B)** Serum cTnI concentration measured by ELISA. **(C)** Serum propylene glycol (PG) concentration measured by GC-MS. **(D)** Relative mRNA expression levels of STAT3 and SLC5A1 measured by RT-qPCR. *p<0.05; **p<0.01; ***p<0.001; ****p<0.0001.

Serum IL-6 and CTnI concentrations were measured by ELISA, as shown in **Figures 9A, B**. Serum IL-6 concentrations were significantly higher in the SCM group than in both the Sepsis and Control groups, while IL-6 in the Sepsis group was significantly higher than in the Control group (P < 0.05). Similarly, cTnI levels reflecting myocardial injury were highest in the SCM group, significantly exceeding those in the Sepsis and Control groups. The difference between the Sepsis and Control groups was also statistically significant (P < 0.05).

Serum propylene glycol concentrations were measured by GC-MS, as shown in **Figure 9C**. Serum propylene glycol was significantly higher in the Control group than in both the Sepsis and SCM groups (P < 0.05). Similarly, serum propylene glycol concentration in the Sepsis group was significantly higher than in the SCM group (P < 0.05).

The expression levels of the biomarker in clinical samples were assessed using RT-qPCR. According to the RT-qPCR results, STAT3 expression was significantly upregulated in both the SCM and Sepsis groups, with the SCM group exhibiting higher expression levels than the Sepsis group (**Figure 9D**) (both P < 0.05). In contrast, SLC5A1 expression exhibited a descending trend across the three groups: highest in the Control group, intermediate in the Sepsis group, and lowest in the SCM group. The difference between the SCM and Control groups was statistically significant (P < 0.05). Furthermore, due to the limited sample size, this clinical validation result is only an exploratory finding and still requires further confirmation in large-scale studies.

## 4 Discussion

SCM, a prevalent complication in sepsis patients, is closely associated with the high mortality rate in these patients. Early diagnosis and effective treatment of SCM are crucial for reducing mortality in sepsis patients. The present study investigated the potential mechanisms by which GM metabolites act on SCM through the use of MR and transcriptomics validation. Initially, MR analysis identified five gut microbiota species that were causally associated with sepsis, among which Bifidobacterium exhibited a significant causal relationship with sepsis. Following a thorough evaluation of multiple machine learning methods, STAT3 and SLC5A1 were identified as the optimal biomarkers for this study. GSEA results demonstrated that both biomarkers exhibited enrichment in myocardial contraction, branched-chain amino acid degradation, oxidative phosphorylation, and neurodegenerative disease pathways. The outcomes of the investigation into the regulatory network involving miRNA-mRNA and TF-mRNA revealed that transcription factors, including FOXC1, FOXL1, and YY1, along with specific miRNAs such as miR-3120-3p, have regulatory interactions with biomarkers. Concurrently, propylene glycol was identified as a promising metabolite through metabolite drug similarity and toxicity assessment. Subsequent molecular docking and molecular dynamics simulations indicated that the two exhibit a certain degree of binding affinity, providing a reference for subsequent in-depth investigation into the specific role of gut microbiota metabolites in SCM. However, these findings still require validation through further experimental studies.

The results of the MR analysis identified the following taxonomic groups: genus.Actinobacteria.id.419, family.Bifidobacteriaceae.id.433, genus.Bifidobacterium.id.436.scatter, order.Bifidobacteriales.id.432.scatter, and phylum.Actinobacteria.id.400. These taxonomic groups were identified as being causally associated with sepsis and as protective factors for sepsis. Bifidobacteria are Gram-positive, anaerobic bacteria that primarily reside in the gastrointestinal tract of animals. They are generally beneficial to humans, particularly in alleviating gastrointestinal, immunological, and infectious diseases. The abundance of bifidobacteria is frequently utilized as a predictor of human health status (34). According to the findings of existing research, the presence of bifidobacteria has been demonstrated to reduce the incidence of sepsis and improve the prognosis of sepsis patients by strengthening intestinal integrity and restoring microbial balance. This effect is achieved through mechanisms such as blocking the NF-κB signaling pathway, regulating the AHR/NRF2/NLRP3 inflammasome pathway, and regulating immunity (35). Intestinal inflammation has been proven to impair the host’s innate immune response and lead to a reduction in bifidobacteria in the gastrointestinal tract. The supplementation of bifidobacteria alone has been demonstrated to reduce mortality in a mouse model of sepsis (36). A body of research has emerged regarding the gut microbiota and metabolites in sepsis models, with studies on adult sepsis patients in intensive care units (37, 38), pediatric sepsis patients (39), and sepsis mouse models (40) yielding consistent results. These studies have revealed significant alterations in the gut microbiota and metabolites in sepsis models, characterized by a reduction in bifidobacteria and beneficial short-chain fatty acids. This finding is consistent with the results of our study, in which MR identified key gut microbiota, such as Bacteroides, as protective factors against sepsis. However, the causal inferences drawn from our study are based on GWAS data for sepsis rather than SCM, and are intended to provide an initial exploration of potential common pathogenic mechanisms from the perspective of gut microbiota. Further validation of this association in SCM-specific cohorts is required.

The findings of the present study show a significant causal relationship between bifidobacteria and sepsis, and previous studies support this result. Consequently, we identified bifidobacteria as a pivotal microorganism, obtained its metabolites, and predicted targets. We then intersected bifidobacteria-related targets with sepsis-related myocardial disease differential genes to obtain 11 candidate genes. By employing a combination of enrichment analysis techniques, including DO, GO, and KEGG, we sought to elucidate the biological processes and signaling pathways that are subject to regulation by the candidate genes implicated in SCM. GO analysis revealed the synchronous enrichment of candidate genes in glucose homeostasis (BP) and cytokine upregulation (BP), suggesting a metabolic-immune crosstalk mechanism. The localization of the apical membrane (CC) is associated with microenvironment sensing functions, while the enrichment of the perinuclear endoplasmic reticulum (CC) is consistent with protein secretion and stress response. These biological processes suggest that under metabolic or inflammatory stress, activation of apical sensors in intestinal epithelial cells triggers an inflammatory response in the perinuclear endoplasmic reticulum, potentially leading to a metabolic disorder (glucose metabolism)-endoplasmic reticulum stress-cytokine release inflammatory mechanism. KEGG enrichment analysis revealed the synergistic effects of the AGE-RAGE signaling pathway, insulin resistance, and chemokine receptor activation in diabetic complications, further validating the metabolic-immune crosstalk mechanism obtained from GO analysis. AGE-RAGE has been observed to trigger persistent oxidative stress and inflammation through the activation of pivotal inflammatory pathways, including NF-κB and JNK (41). Persistent metabolic disorders induce ROS bursts, which have the potential to exacerbate septic cardiomyopathy (42). DO enrichment reveals associations between gastric cancer, primary immunodeficiency, and chemical carcinogenesis, with inflammation being a common pathogenic mechanism in these three diseases. Metabolic disorders, including hyperglycemia and insulin resistance, activate chronic inflammation through the AGE-RAGE pathway. This activation is further amplified by immune deficiency, resulting in an intensified inflammatory response. The above functional analyses suggest that the GM may be involved in the development of SCM through metabolic-immune crosstalk mechanisms.

Through a series of machine learning methods, signal transducer and activator of transcription 3 (STAT3) and Solute Carrier Family 5 Member 1 (SLC5A1) were ultimately identified as key biomarkers. ROC curve analysis results demonstrated that these two genes exhibited excellent predictive performance for SCM. STAT3 is a nuclear transcription factor. It is currently known to regulate biological processes such as embryonic development, innate and adaptive immunity, cell growth and differentiation, and programmed cell death. Some researchers have implicated this gene in ischemic heart disease (43). In SCM multiple studies combining bioinformatics analysis methods with mouse cell validation have identified STAT3 as a key biomarker for SCM, and it may be associated with ferroptosis (44, 45). With regard to the mechanisms involved, research has determined that microRNA-206 directly targets USP33, thereby impeding the JAK2/STAT3 signaling pathway and effectively mitigating the inflammatory damage to myocardial cells in septic mice (46), melatonin can also alleviate myocardial damage in septic mice by regulating the JAK2/STAT3 signaling pathway (47). Traditional Chinese medicine treatment involving electroacupuncture can protect the hearts of septic mice from inflammation and cell apoptosis by inhibiting the calpain-2/STAT3 pathway (48). It has been clearly confirmed that our research results are consistent with previous related findings.

SLC5A1, also named Na⁺/Glucose Cotransporter 1 (SGLT1), is a gene that encodes a sodium-dependent glucose transporter (SGLT). It is primarily expressed in intestinal epithelial cells and in the S3 segment of proximal tubules in the kidneys. SLC5A1 plays a key role in metabolism by promoting glucose absorption in the small intestine and influencing intestinal microbiota composition (49). A cohort study found that inhibiting SGLT1 improves subclinical left ventricular ejection fraction dysfunction and may prevent heart failure (50). SGLT1 inhibitors are currently approved to treat heart failure (51), SGLT1 has become a key player in cardiac function due to its important role in glucose metabolism and cardiac energy supply (52). Researchers found that inhibiting SGLT1 can improve ischemia-reperfusion-induced cardiac damage in an ischemia-reperfusion mouse model (53). Further studies using diabetic mouse models revealed that SGLT1 can damage cardiomyocytes by activating the NLRP3/caspase-1 and NF-κB pathways (54). Inhibiting SGLT1 can reduce apoptosis of myocardial cells caused by glucose fluctuations induced by oxidative stress and mitochondrial dysfunction (55). Previous studies on the SGLT1 mechanism have primarily examined diabetic mouse models, which have demonstrated its role in promoting myocardial damage during blood glucose fluctuations. Cohort studies in humans have also revealed that SGLT1 may impact cardiac function via glucose metabolism and energy supply. There are currently no studies on the relationship between SGLT1 and SCM, but its close relationship with the heart supports further investigation into its relationship with the pathogenesis of SCM.

GSEA enrichment analysis revealed that the two biomarkers were predominantly enriched in pathways associated with myocardial contraction, branched-chain amino acid degradation, oxidative phosphorylation, and neurodegenerative disease. Previous studies have demonstrated that the gut microbiota plays a role in the microbiome-gut-brain axis by influencing neurological function through mechanisms such as immunity, short-chain fatty acids, and Branched-chain amino acids(BCAAs). This study is of considerable significance for neurodegenerative diseases including Alzheimer’s and Parkinson’s (56, 57). BCAAs are found in the periphery and include essential amino acids such as leucine, isoleucine, and valine. The metabolic end products of these amino acids are short-chain fatty acids, which can protect dopaminergic neurons by inhibiting overall inflammatory responses. This improves symptoms in mice with Parkinson’s disease (58). Research in the field of cardiovascular health has found a connection between the enhancement of BCAAs catabolism and the prevention of cardiovascular damage (59), reducing levels of BCAAs may improve outcomes for patients with heart failure (60). A cohort study found a positive correlation between BCAAs and cardiovascular event incidence (61). Researchers studying a mouse model of type 1 diabetes have found that excessive BCAAs cause mitochondrial damage and myocardial apoptosis through the mTOR signaling pathway, leading to cardiac fibrosis and dysfunction in T1D mice (62). Oxidative stress can impact the contractile function of the mouse heart (63). In obese aged mouse models, researchers found that STAT3 can inhibit phosphorylation and regulate reactive oxygen. These can participate in regulating cardiac remodeling and myocardial contractile dysfunction (64). After undergoing various oxidative stress modifications, STAT3 can act as an intermediary for metabolism and mitochondrial activity (65). IL-6 regulates energy metabolism by acting through two pathways: the PDK1/STAT3/PDHA axis and the SIRT2/STAT3/LDHA axis. These pathways allow IL-6 to exert both anti-inflammatory and pro-inflammatory functions (66). Based on previous studies and the enriched pathways we obtained, we can speculate that: STAT3 may influence myocardial contractile function in SCM by inhibiting calmodulin and oxidative phosphorylation and disrupting mitochondrial biogenesis, SLC5A1 may affect myocardial cell metabolism through branched-chain amino acids. These factors may contribute to a vicious cycle of metabolic disorders, impaired myocardial contractility, and exacerbated inflammation, which ultimately leads to myocardial cell dysfunction in sepsis.

The main feature of SCM is immune-mediated damage to the heart muscle, and the immune response plays a key role in its development (67). Our analysis of immune infiltration revealed that STAT3 was positively correlated with activated CD4+ T cells, activated dendritic cells, CD56dim natural killer cells, immature dendritic cells, myeloid-derived suppressor cells (MDSCs), monocytes, natural killer T cells, neutrophils, and plasmacytoid dendritic cells. STAT3 was negatively correlated with type 2 T helper cells. SLC5A1 showed the opposite correlation. This result clearly shows that innate and adaptive immunity both play important roles in sepsis-associated cardiomyopathy. Dendritic cells are instrumental in integrating the functions of both innate and adaptive immunity (68). Dendritic cells control the activation and maintenance of various CD4+ T helper subsets and CD8+ cytotoxic T cell responses. They differentiate into different lineages by utilizing glucose in various metabolic microenvironments (69). The reduced number of dendritic cells in patients with sepsis is also a reason for the high mortality rate (70). In a sepsis cell model, researchers found that microRNA-146a-5p (miR-146a-5p) can target autophagy-related gene 7 (ATG7) to regulate the levels of STAT3 phosphorylation, which is a signal sensor and activator. This process exacerbates sepsis by increasing dendritic cell activation and glycolysis (71). These findings suggest that, through the regulation of STAT3 and SLC5A1 in the intestinal microbiota metabolic environment, dendritic cells may exert anti-inflammatory or pro-inflammatory effects, thereby influencing sepsis-induced multiple organ dysfunction.

After constructing the molecular regulatory network, we discovered regulatory relationships between the two biomarkers we screened and transcription factors such as Forkhead Box C1 (FOXC1), Forkhead Box L1 (FOXL1), YY1, and microRNA-3120-3p. FOXC1 can regulate cell migration. Studies have shown that overexpressing FOXC1 inhibits the migration of microglia while regulating the IκBα/NF-κB pathway, which suppresses inflammatory responses and neuronal apoptosis. This improves cognitive dysfunction in patients with sepsis (72). FOXL1 may be related to cell development. Studies have found that FOXL1 participates in the development of the intestinal mucosal structure and function. It can also increase the risk of neonatal sepsis through intestinal microbial translocation (73). YY1 participates in the inhibition and activation of different numbers of promoters and can participate in NF-kB, IL-6, IL-10, and TLR signal transduction, participate in leukocyte transcription, and affect the prognosis of patients with sepsis (74). YY1 can improve the prognosis of patients with sepsis-associated encephalopathy by promoting the polarization of microglia to the M2 state (75). Furthermore, YY1 has the potential to avert multiple organ dysfunction in children with sepsis by modulating apoptosis and can function as a prognostic indicator for children with sepsis (76).miR-3120-3p is associated with STAT3 and SLC5A1. It is a potential biomarker for head and neck epithelial cell carcinoma and may be related to nerve growth and bacterial invasion. It can cause cognitive impairment by influencing the development of the intestinal microbiota (77). In the course of our analysis of molecular regulatory networks, we ascertained that transcription factors such as FOXC1, FOXL1, YY1 and miR-3120-3p, may be associated with the regulation of biomarkers: Among these, FOXL1 controls intestinal mucosal growth, while YY1 takes part in inflammatory pathways by regulating promoters, thus affecting the transcription of miR-3120-3p and connecting the GM, inflammatory reaction, sepsis, and sepsis-related organ dysfunction. This finding suggests the possibility of a link between the GM and cardiovascular health, via these molecular associations. The results provide preliminary evidence to support further research into the potential consequences of these associations.

In the metabolite prediction results, we found that Propylene Glycol is a promising metabolite. Molecular docking and molecular dynamics results confirmed that this metabolite binds well to biomarkers. These findings indicate that metabolites associated with this biomarker may possess research value in SCM; however, further experimental evidence is required to clarify their specific role and therapeutic implications. Propylene Glycol is a common ingredient in topical pharmaceuticals and has antibacterial effects against the bacterium Staphylococcus aureus (78). A study of dairy cows found that supplementing their diet with BCAAs and Propylene Glycol reduced the accumulation of lipids in their livers (79). This may suggest that Propylene Glycol exerts its effects by promoting BCAAs, which is also related to the influence of GM on the degradation of BCAAs on myocardial cell apoptosis and contraction in mice, as we talked about before. The utilization of liposomes, which are loaded with propylene glycol as a pharmaceutical agent, has been demonstrated to play a pivotal role in the prevention of ischemic cerebrovascular disease, particularly in patients diagnosed with diabetes (80). In a mouse model of ulcerative colitis, the combination of anti-inflammatory agents and the drug demonstrated effective mitigation of the progression of inflammation and symptoms, enhanced inhibition of pro-inflammatory cytokine secretion (such as IL-6 and TNF-α), and augmented therapeutic effects (81). These findings suggest that future research could focus further on the potential applications of propylene glycol in SCM, either as a candidate metabolite or as a carrier component in drug delivery systems. However, its precise role still requires verification through further experimentation.

To validate the reliability of the aforementioned analytical results, this study further collected 27 clinical samples. Using ELISA, GC-MS, and RT-qPCR techniques, preliminary detection was conducted on key inflammatory factors, myocardial injury markers, predictive metabolites, and biomarker gene expression levels. The preliminary clinical sample validation results showed high consistency with the earlier bioinformatics analysis predictions based on public databases. The expression patterns of STAT3 and SLC5A1, in conjunction with alterations in propylene glycol metabolism, imply that these factors may contribute to the pathological process of SCM. To a certain degree, these findings offer cross-validation of the feasibility of screening for biomarkers and investigating underlying mechanisms from the perspective of the gut microbiota and its metabolites. However, given the limited size of the clinical sample in this study and the fact that only mRNA expression levels were measured, the above conclusions require further confirmation in a larger, independent cohort, in conjunction with functional experiments.

The objective of this study is to use bioinformatics methodologies to investigate the potential correlation between GM metabolites and SCM. The integration of GM metabolite data from public databases with myocardial tissue transcriptomic information has enabled the screening and identification of STAT3 and SLC5A1 as potential disease-associated biomarkers. Concurrently, the biological pathways potentially implicated, including inflammatory responses and amino acid degradation, were analysed, and the binding activity of propylene glycol as a candidate metabolite was predicted. These findings provide a foundation for understanding the molecular characteristics of SCM, and the identified genes and pathways may serve as a reference for subsequent in-depth mechanistic studies and the exploration of potential intervention targets. While these findings are of potential significance, it must be noted that the conclusions drawn thus far are primarily based on inference and require further confirmation through functional experimentation.

Nevertheless, it must be acknowledged that our research is not without its limitations. First, the study’s starting point relied on GWAS data from sepsis rather than SCM to infer causal relationships between gut microbiota and disease. While this limitation stems from data availability, it may have resulted in the omission of certain more strongly associated specific microbiota or targets. Second, the clinical trial sample size was limited and not formally estimated, which reduced statistical power and the generalisability of the results. Furthermore, the employment of feature selection algorithms carries a potential risk of overfitting. The assessment of the general applicability and diagnostic efficacy of STAT3 and SLC5A1 as biomarkers, as currently screened, may be limited by sample size and influenced by the inherent biases of the feature selection algorithm. In future work, we plan to establish an independent external validation cohort through the prospective collection of multicentre clinical samples, and to further evaluate the diagnostic stability and clinical utility of these two biomarkers through relevant in-depth experiments. The study involved the analysis of transcriptomic data from public databases. It lacked in-depth experimental studies to validate the underlying mechanisms, and the validation of biomarkers was limited to the mRNA level, with no confirmation at the protein or functional level. Consequently, contemporary mechanistic conclusions are primarily based on inference. Further research is necessary to collect SCM-specific cohort data, expand the sample size, and conduct *in vivo* and *in vitro* functional experiments alongside multi-centre validation. These studies will elucidate the specific regulatory mechanisms and clinical value of the gut microbiota and its metabolites in SCM.

## 5 Conclusion

The present study explored the association between gut microbial metabolites and SCM by integrating MR and transcriptomic analysis. STAT3 and SLC5A1 were identified as candidate biomarkers with potential discriminatory power, and propylene glycol was found to be a relevant candidate metabolite. As these findings are primarily based on exploratory analyses, their precise value in clinical diagnosis and treatment requires further in-depth validation.

## Data Availability

The original contributions presented in the study are included in the article/**Supplementary Material**. Further inquiries can be directed to the corresponding author.

## Supplementary material

Supplementary Figure 1 Scatter plot of Mendelian analysis. **(A)** class.Actinobacteria.id.419. **(B)** family.Bifidobacteriaceae.id.433. **(C)** genus.Bifidobacterium.id.436. **(D)** order.Bifidobacteriales.id.432. **(E)** phylum.Actinobacteria.id.400.

Supplementary Figure 2 Forest plots of Mendelian analysis. **(A)** class.Actinobacteria.id.419. **(B)** family.Bifidobacteriaceae.id.433. **(C)** genus.Bifidobacterium.id.436. **(D)** order.Bifidobacteriales.id.432. **(E)** phylum.Actinobacteria.id.400.

Supplementary Figure 3 Funnel plot of Mendelian analysis. **(A)** class.Actinobacteria.id.419. **(B)** family.Bifidobacteriaceae.id.433. **(C)** genus.Bifidobacterium.id.436. **(D)** order.Bifidobacteriales.id.432. **(E)** phylum.Actinobacteria.id.400.

Supplementary Figure 4 Leave-one-out test forest plots for Mendelian analysis. **(A)**class.Actinobacteria.id.419. **(B)** family.Bifidobacteriaceae.id.433. **(C)** genus.Bifidobacterium.id.436. **(D)** order.Bifidobacteriales.id.432. **(E)** phylum.Actinobacteria.id.400.

Supplementary Figure 5 Validation of key findings after multiple comparison correction. **(A)** GO enrichment analysis results after BH correction. **(B)** KEGG enrichment analysis results after BH correction. **(C)** DO enrichment analysis results after BH correction. **(D)** GSEA enrichment plot for SLC5A1 after BH correction. **(E)** GSEA enrichment plot for STAT3 after BH correction. **(F)** Heatmap of relative abundance of 28 immune cell types. **(G)** Box plot of immune cell infiltration abundance between SCM and control groups after BH correction. **(H)** Heatmap of Spearman correlation between key biomarkers (STAT3 and SLC5A1) and differential immune cells after BH correction. **(I)** Correlation network among differential immune cells after BH correction. *, p<0.05; **p<0.01; ***p<0.001; ****p<0.0001.

Supplementary Table 1 Baseline clinical characteristics of enrolled participants.

Supplementary Table 2 Primer sequences used for RT-qPCR.

Supplementary Table 3 Metabolites and targets corresponding to genus.Bifidobacterium.id.436.scatter.

Supplementary Table 4 Enriched GO terms for candidate genes in SCM.

Supplementary Table 5 Enriched KEGG pathways for candidate genes in SCM.

Supplementary Table 6 Enriched DO terms for candidate genes in SCM.

Supplementary Table 7 GSEA results of STAT3 and SLC5A1 in SCM.

Supplementary File 1 Analysis code used in this study.
