# Supplementary figures and images for: The mechanism of gut microbiota in septic cardiomyopathy based on the bulk transcriptome and Mendelian randomization analysis
Source: Front Immunol. 2026 May 4;17:1799675. doi: 10.3389/fimmu.2026.1799675 (PMC13181340; doi:10.3389/fimmu.2026.1799675)

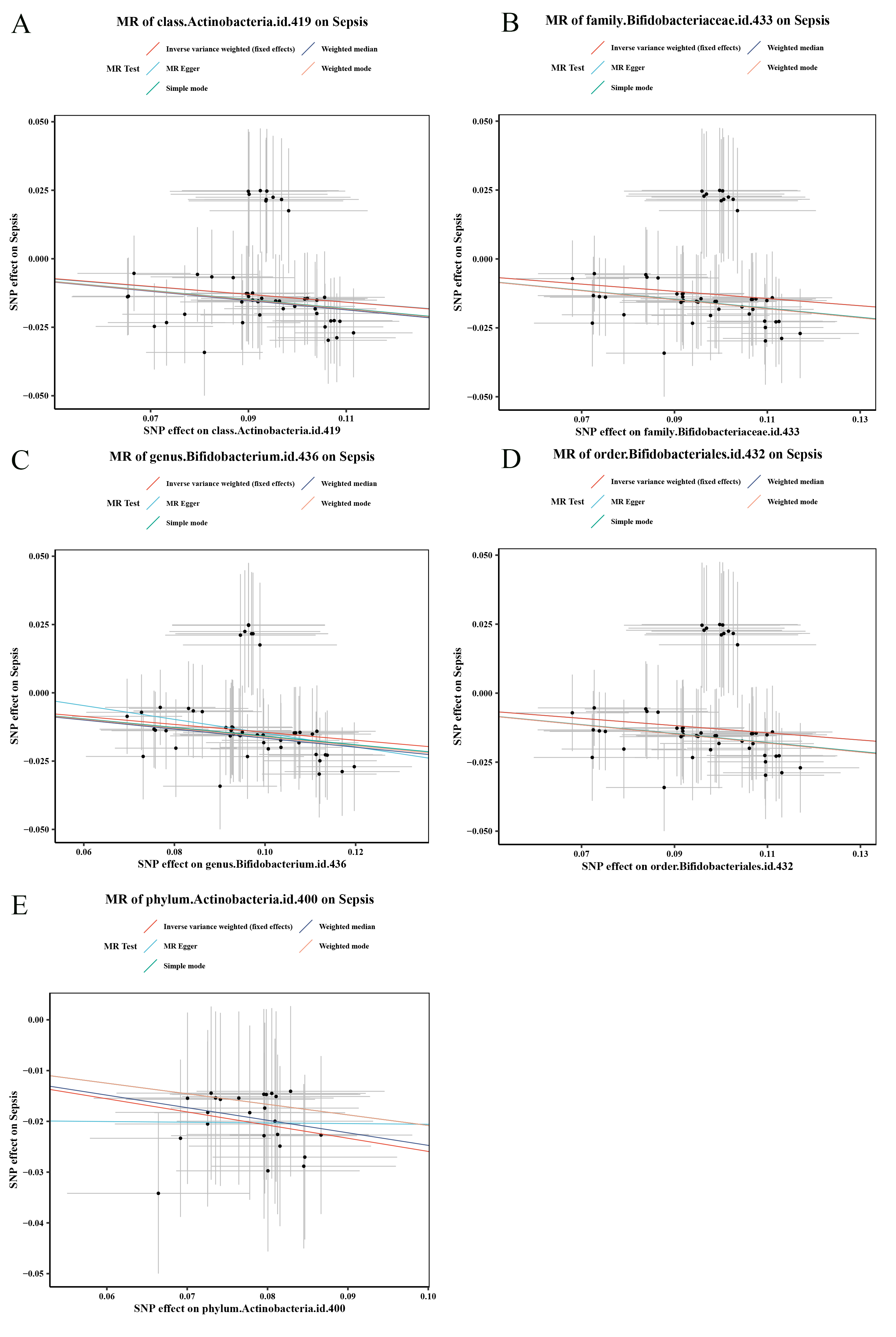

Supplement: Supplementary Figure 1 — Scatter plot of Mendelian analysis. (A) class.Actinobacteria.id.419. (B) family.Bifidobacteriaceae.id.433. (C) genus.Bifidobacterium.id.436. (D) order.Bifidobacteriales.id.432. (E) phylum.Actinobacteria.id.400. [file Image1.jpeg]

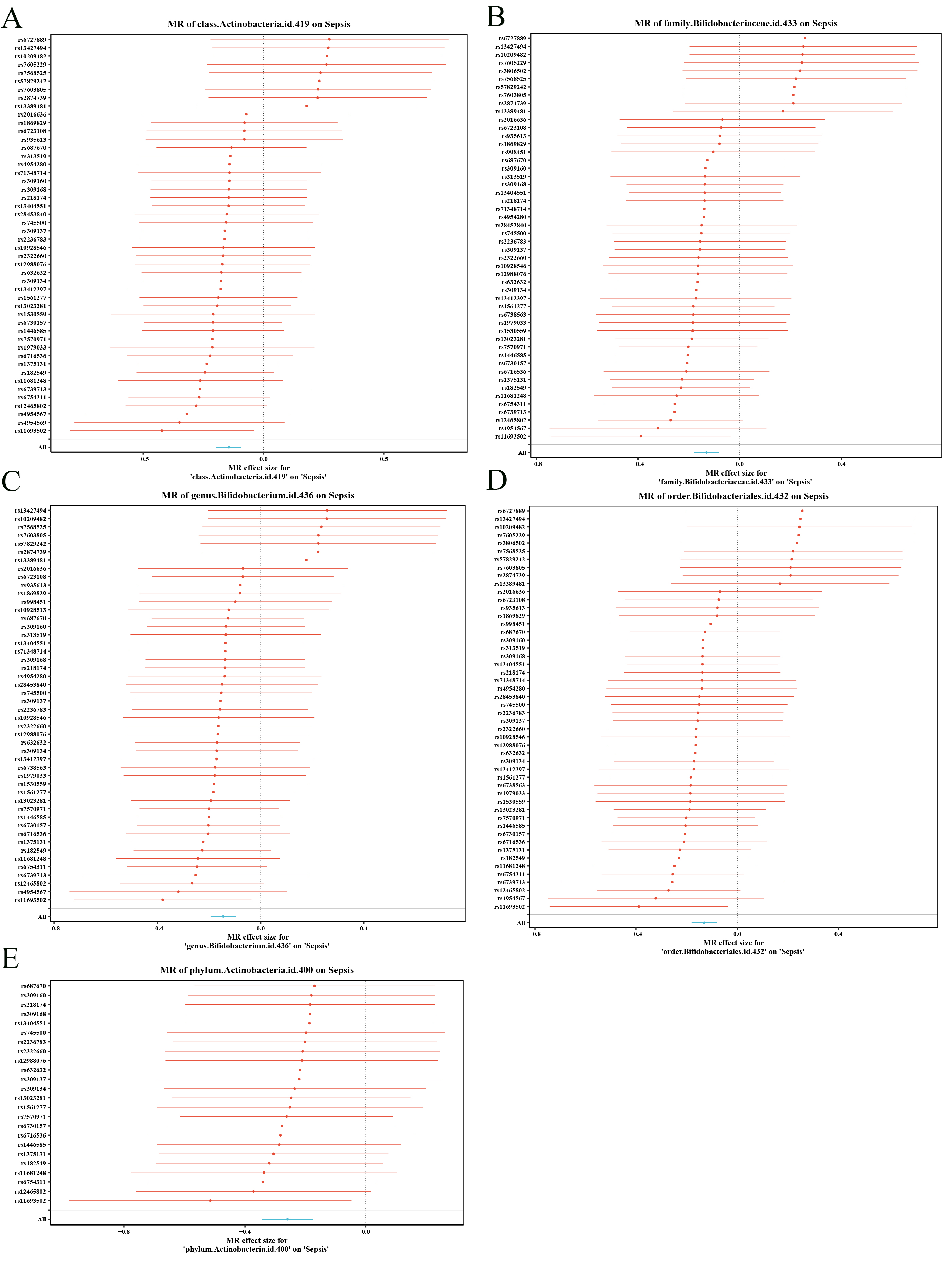

Supplement: Supplementary Figure 2 — Forest plots of Mendelian analysis. (A) class.Actinobacteria.id.419. (B) family.Bifidobacteriaceae.id.433. (C) genus.Bifidobacterium.id.436. (D) order.Bifidobacteriales.id.432. (E) phylum.Actinobacteria.id.400. [file Image2.jpeg]

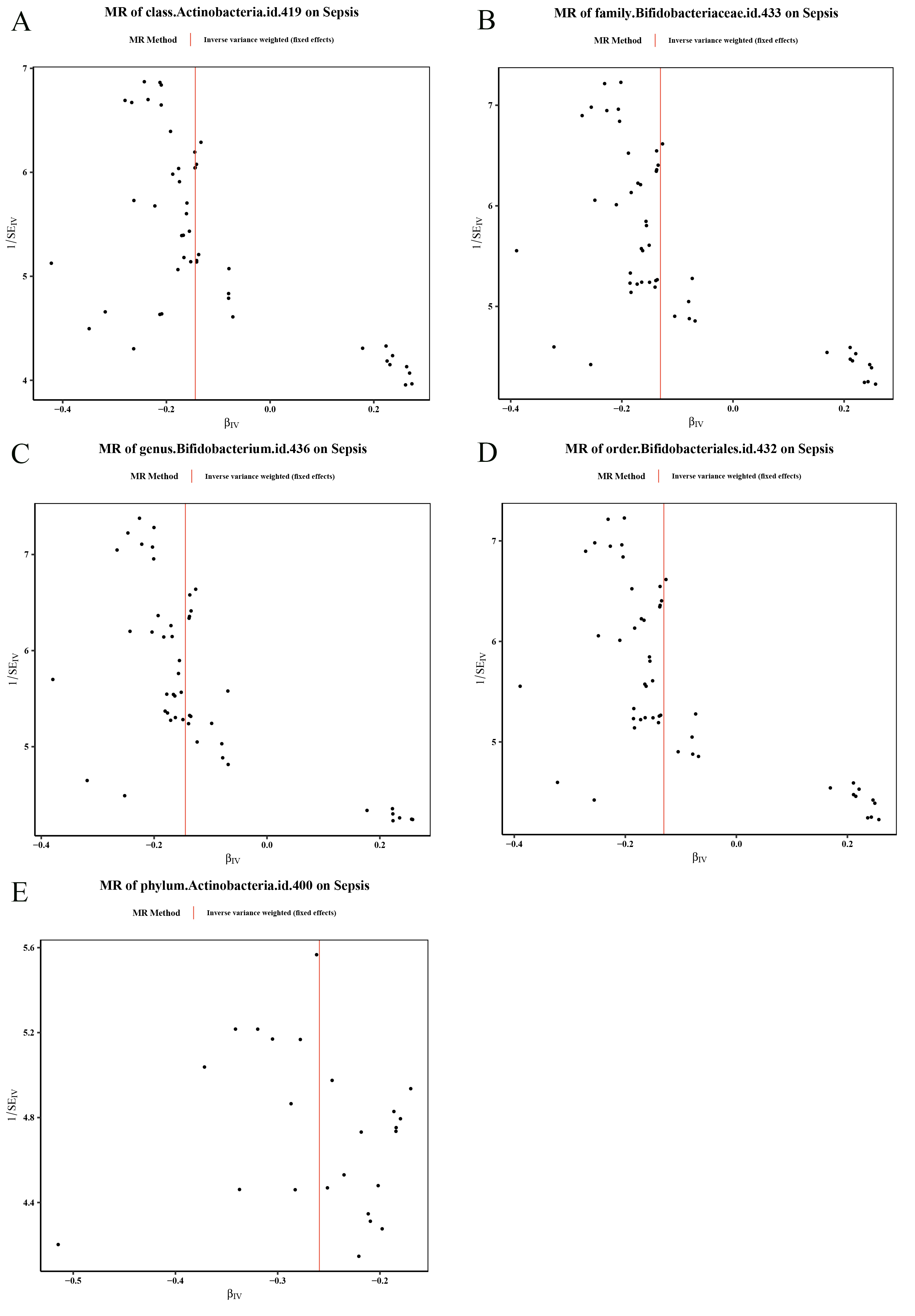

Supplement: Supplementary Figure 3 — Funnel plot of Mendelian analysis. (A) class.Actinobacteria.id.419. (B) family.Bifidobacteriaceae.id.433. (C) genus.Bifidobacterium.id.436. (D) order.Bifidobacteriales.id.432. (E) phylum.Actinobacteria.id.400. [file Image3.jpeg]

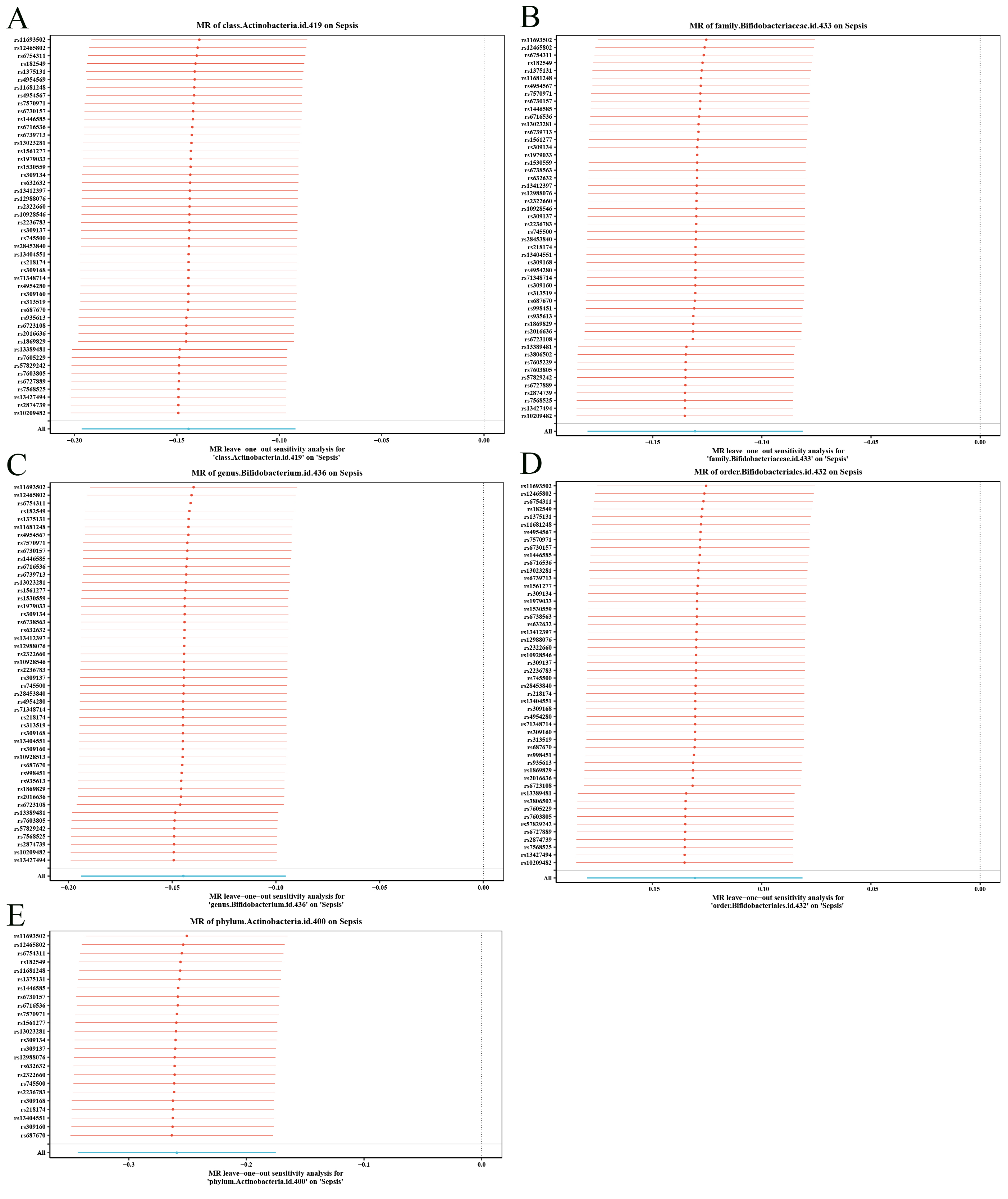

Supplement: Supplementary Figure 4 — Leave-one-out test forest plots for Mendelian analysis. (A)class.Actinobacteria.id.419. (B) family.Bifidobacteriaceae.id.433. (C) genus.Bifidobacterium.id.436. (D) order.Bifidobacteriales.id.432. (E) phylum.Actinobacteria.id.400. [file Image4.jpeg]

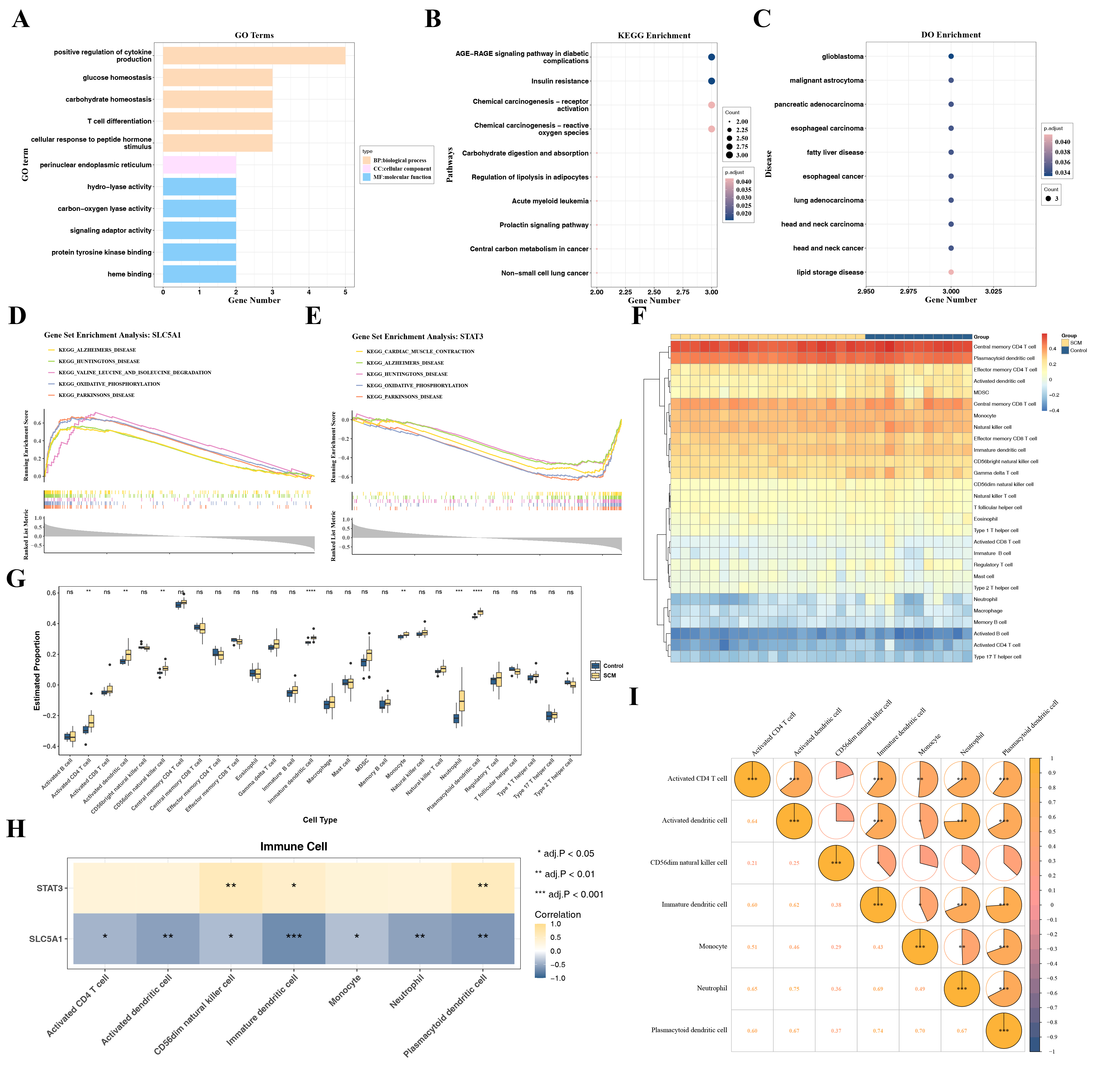

Supplement: Supplementary Figure 5 — Validation of key findings after multiple comparison correction. (A) GO enrichment analysis results after BH correction. (B) KEGG enrichment analysis results after BH correction. (C) DO enrichment analysis results after BH correction. (D) GSEA enrichment plot for SLC5A1 after BH correction. (E) GSEA enrichment plot for STAT3 after BH correction. (F) Heatmap of relative abundance of 28 immune cell types. (G) Box plot of immune cell infiltration abundance between SCM and control groups after BH correction. (H) Heatmap of Spearman correlation between key biomarkers (STAT3 and SLC5A1) and differential immune cells after BH correction. (I) Correlation network among differential immune cells after BH correction. *, p<0.05; **p<0.01; ***p<0.001; ****p<0.0001. [file Image5.jpeg]
